# Ionic Liquids with More than One Metal: Optical and Electrochemical Properties versus d‐Block Metal Combinations

**DOI:** 10.1002/chem.202003097

**Published:** 2020-12-03

**Authors:** Christian Balischewski, Karsten Behrens, Kerstin Zehbe, Christina Günter, Stefan Mies, Eric Sperlich, Alexandra Kelling, Andreas Taubert

**Affiliations:** ^1^ Institute of Chemistry University of Potsdam 14476 Potsdam Germany; ^2^ Institute of Geosciences University of Potsdam 14476 Potsdam Germany

**Keywords:** bandgap, electrochemistry, ionic liquids, metal-containing ionic liquids, tetrahalido metallates

## Abstract

Thirteen *N*‐butylpyridinium salts, including three monometallic [C_4_Py]_2_[MCl_4_], nine bimetallic [C_4_Py]_2_[M_1−*x*_^a^M_*x*_^b^Cl_4_] and one trimetallic compound [C_4_Py]_2_[M_1−*y*‐*z*_^a^M_*y*_^b^M_*z*_^c^Cl_4_] (M=Co, Cu, Mn; *x=*0.25, 0.50 or 0.75 and *y*=*z=*0.33), were synthesized and their structure and thermal and electrochemical properties were studied. All compounds are ionic liquids (ILs) with melting points between 69 and 93 °C. X‐ray diffraction proves that all ILs are isostructural. The conductivity at room temperature is between 10^−4^ and 10^−8^ S cm^−1^. Some Cu‐based ILs reach conductivities of 10^−2^ S cm^−1^, which is, however, probably due to IL dec. This correlates with the optical bandgap measurements indicating the formation of large bandgap semiconductors. At elevated temperatures approaching the melting points, the conductivities reach up to 1.47×10^−1^ S cm^−1^ at 70 °C. The electrochemical stability windows of the ILs are between 2.5 and 3.0 V.

## Graphical abstract

**Mixed metal** tetrahalidometallate ionic liquids (ILs) were for the first time synthesized and studied for their structural, optical, and electrochemical properties. The ILs exhibit high conductivities and tunable optical absorption and are thus novel and highly versatile candidates for application in electronic and optical devices.

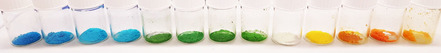

## Introduction

Ionic liquids (ILs) have become a popular research field over the last decades. This is due to their interesting and often rather unusual properties. ILs are salts with melting points below ca. 100 °C (although this temperature is arbitrary), which is much lower than those of conventional (inorganic) salts. They often display high ionic conductivity and sometimes even good electronic conductivity and often have a broad electrochemical stability window. At the same time they are also good solvents for inorganic and organic compounds.[^1^, ^2^, ^3^, ^4^] As a consequence ILs have a high application potential and are often used as electrolytes in batteries, fuel cells, actuators, and photovoltaic cells.[^5^, ^6^, ^7^, ^8^] Moreover, ILs are typically safe and easy to handle. This is largely due to their low vapor pressure and low flammability. As a result, ILs can often act as a solvent or catalyst.[^4^, ^9^] By altering specific functional groups of an IL or changing the anion or cation, the chemical, physical, or biological properties of the ILs can be adjusted to fit a particular application.[^2^, ^9^] Therefore, ILs are generally interesting materials for a number of applications, despite the fact that they are hygroscopic and have a high viscosity.

A particularly interesting IL subclass is the group of metal‐containing ILs (MILs).^[10]^ MILs are ILs with at least one metal ion in the IL anion or cation. Consequently, MILs have interesting electrochemical, magnetic, catalytic, and optical properties, which cannot be obtained otherwise.[^3^, ^9^, ^11^, ^12^, ^13^, ^14^, ^15^, ^16^, ^17^, ^18^, ^19^, ^20^, ^21^] Especially transition‐metal‐based MILs have attracted attention. This is, among others, due to the fact MILs are not only interesting materials in their own right but can also act as ionic liquid precursors (ILPs) or, as they are also termed, “all‐in‐one solvent‐template‐reactants”,^[22]^ for the controlled synthesis of inorganic and hybrid materials.[^13^, ^22^, ^23^, ^24^, ^25^, ^26^] The combination of transition metals and halides provides access to the [MX_4_]^2−^ (M=Pd, Cu, Co, Mn, Zn, Ni, In, Ga, Cd, etc.; X=Cl, Br, I) unit. If combined with suitable organic cations, these units are the basis for a large group of MILs, the halidometallate ILs, which arguably is the largest subgroup of MILs studied today.[^10^, ^11^, ^27^, ^28^, ^29^, ^30^, ^31^, ^32^, ^33^, ^34^, ^35^, ^36^]

However, although many different metals are in principle amenable to the synthesis of halidometallate ILs, there are still only few reports on MILs containing more than one metal. Very recently, Liu et al.^[37]^ demonstrated mixed metal ILs by combining monometallic ILs with an additional metal salt. The authors further investigated the acidity and basicity of these ILs, as well as different surface parameters. Rogers et al.^[38]^ demonstrated the synthesis of bimetallic ILs from group III metals and transition metals. These articles, however, do not investigate or correlate the electrochemical, optical, or structural properties of bimetallic or trimetallic ILs. Furthermore, a fundamental understanding of the relationships between these aspects is not possible yet.

Consequently, there is essentially no information on the change of the optical, electronic, or magnetic properties upon combination of at least two (transition) metals in the same IL. The current article is, therefore, the first detailed and quantitative report on bi‐ and trimetallic ILs and on their properties, specifically their bandgaps and their conductivities vs. chemical composition. For this initial study we focus on Cu, Co, and Mn‐based ILs, but other transition‐metal‐based ILs are clearly within close reach.

## Results

**Figure 1:**
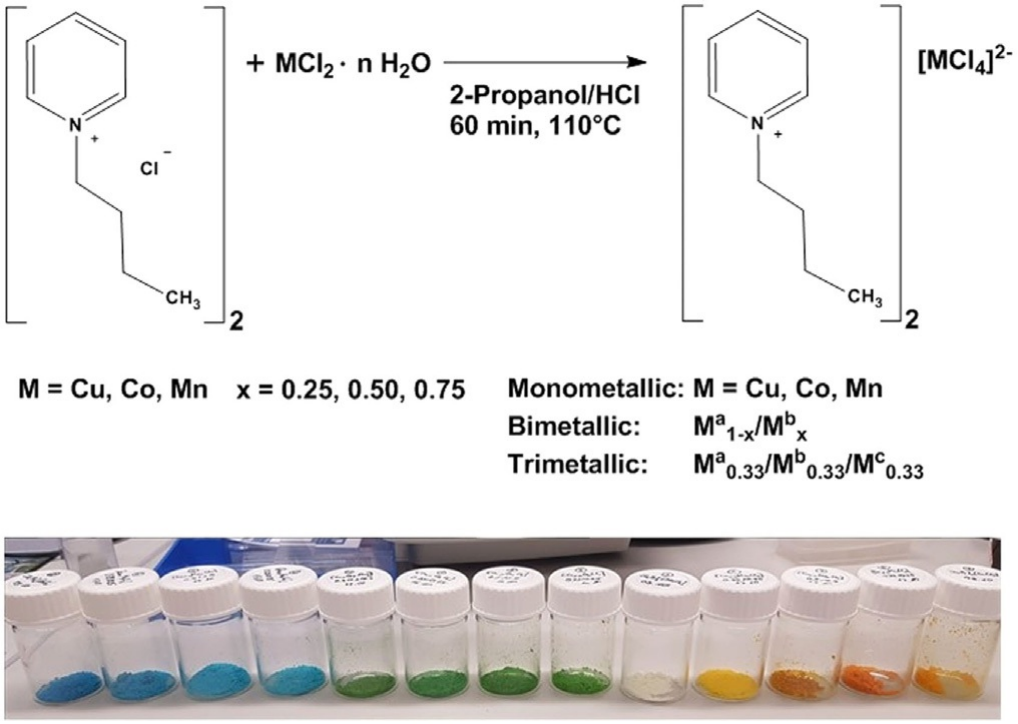
Top: MIL synthesis protocol with M^a^, M^b^, and M^c^ being either Cu, Co or Mn. Bottom: Photo of the solid MILs at room temperature. For image with IL assignments, see electronic Supporting Information (Figure S1).

Synthesis of all compounds was achieved using established and published protocols from commercial starting materials.^[11]^ Figure 1 shows the general synthesis and the resulting ILs. All products are solids at room temperature, but melt between 60 and 93 °C.

The composition of the ILs was analyzed using inductively coupled plasma optical emission spectrometry (ICP‐OES) and elemental analysis (EA). The results are shown in Table 1 and Table S1. Overall, EA (Table S1, Supporting Information) shows that the organic fraction of the different ILs does not differ between the compounds. Furthermore, the analytical data of the ILs do not deviate significantly from the theoretical composition with a maximum discrepancy of 1.5 wt % (with the most significant deviation observed for H, most likely from adsorbed water).

**Table 1:** Metal contents in the ILs determined via ICP‐OES measurements.

Compound (theoretical composition from synthesis)	Abbreviation	Cu [at %]	Co [at %]	Mn [at %]
[C_4_Py]_2_[Cu_0.25_Mn_0.75_Cl_4_]	**IL1**	29.45±0.68	–	70.55±0.68
[C_4_Py]_2_[Cu_0.50_Mn_0.50_Cl_4_]	**IL2**	55.56±0.33	–	44.44±0.33
[C_4_Py]_2_[Cu_0.75_Mn_0.25_Cl_4_]	**IL3**	79.79±0.20	–	20.21±0.20
[C_4_Py]_2_[Cu_0.25_Co_0.75_Cl_4_]	**IL4**	27.68±0.35	72.32±0.35	–
[C_4_Py]_2_[Cu_0.50_Co_0.50_Cl_4_]	**IL5**	50.51±0.13	49.49±0.13	–
[C_4_Py]_2_[Cu_0.75_Co_0.25_Cl_4_]	**IL6**	77.85±0.38	22.15±0.38	–
[C_4_Py]_2_[Co_0.25_Mn_0.75_Cl_4_]	**IL7**	–	26.13±0.65	73.87±0.65
[C_4_Py]_2_[Co_0.50_Mn_0.50_Cl_4_]	**IL8**	–	51.51±1.39	48.49±1.39
[C_4_Py]_2_[Co_0.75_Mn_0.25_Cl_4_]	**IL9**	–	77.32±0.75	22.68±0.75
[C_4_Py]_2_[Cu_0.33_Co_0.33_Mn_0.33_Cl_4_]	**IL10**	37.53±0.55	32.16±0.34	30.31±0.22
[C_4_Py]_2_[CuCl_4_]^[a]^	**IL11**	–	–	–
[C_4_Py]_2_[CoCl_4_]^[a]^	**IL12**	–	–	–
[C_4_Py]_2_[MnCl_4_]^[a]^	**IL13**	–	–	–

[a] ICP‐OES data for the monometallic ILs were not determined due to the fact that no other metals can be expected in these compounds.

ICP‐OES data consistently show a very good agreement between the compositions calculated from the synthesis and the experimentally obtained IL compositions after synthesis. While some ILs, such as **IL1** (theoretical Cu:Mn composition of 25:75) show a deviation of about 5 atom% between the theoretical and real composition, the general discrepancy is under 3 atom% (including an instrument error of 2.5 %), thus resulting in a very good agreement between the composition of the reaction mixture and the resulting solid product. This shows that our synthetic approach is a viable strategy for the synthesis of ILs containing two or three metals in precisely defined ratios.

**Figure 2:**
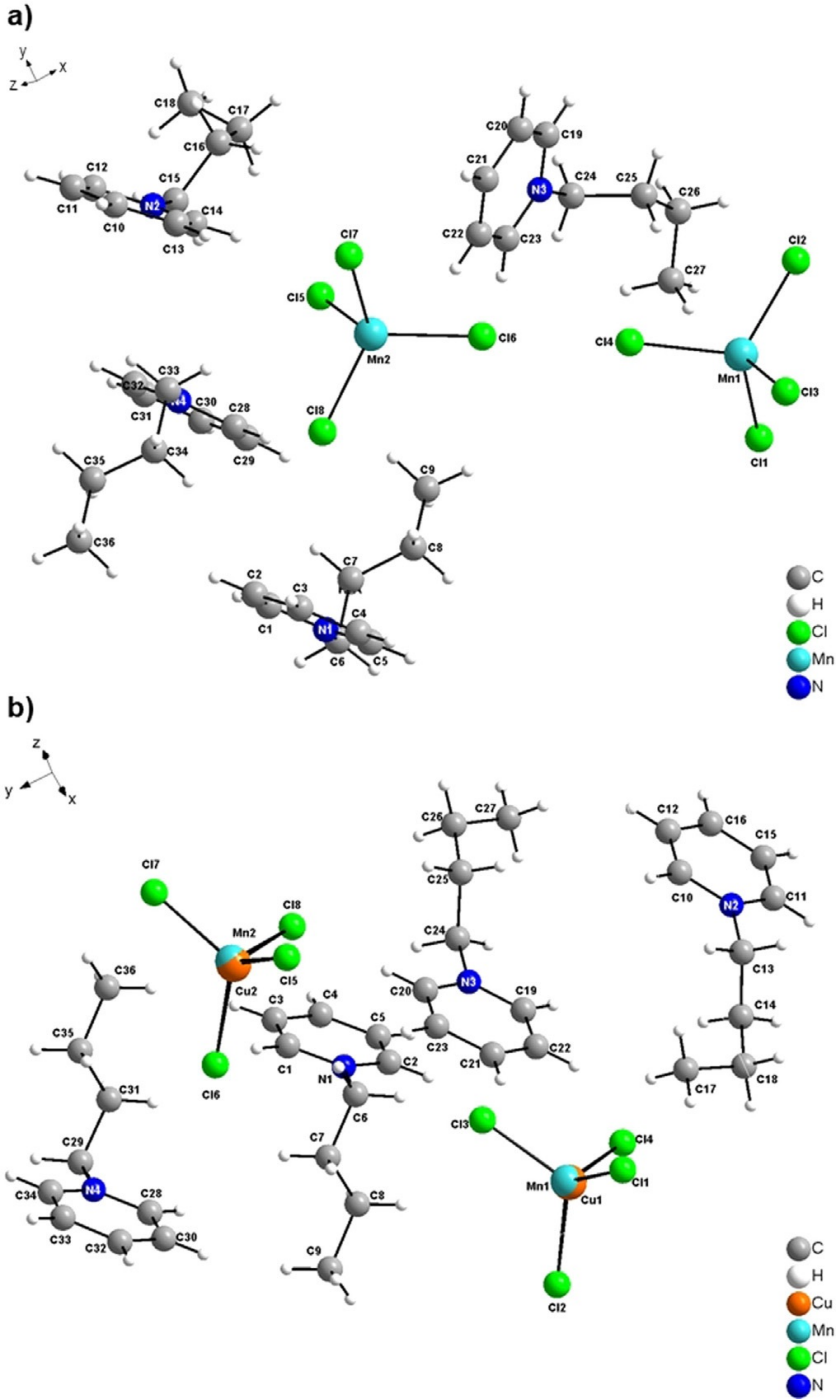
Asymmetric units of a) **IL13** and b) **IL2**. Hydrogen labels are omitted for clarity.

**Figure 3:**
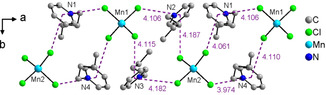
Anion–π interactions between pyridine rings of the cations and chlorido ligands of the anion in **IL 13**.

Figure 2 shows the asymmetric units of **IL2** (b) and **IL13** (a) obtained from single‐crystal X‐ray diffraction. Both ILs (Table 1) crystallize in the monoclinic space group *P*2_1_/*n*, which is consistent with previous work.^[10]^ The asymmetric unit of both ILs contains four *N*‐butylpyridinium cations and two tetrahedrally coordinated tetrachloridometallate(II) anions. Between the pyridine rings of the cations and the chlorido ligands of the anions, so‐called anion–π interactions occur. All symmetry‐independent cations and anions are involved in these interactions, a total number of eight such contacts are observed with a distance of 3.974 Å–4.187 Å (Figure 3). This results in the formation of chains along the crystallographic a‐axis within the crystal structure. Beside these interactions, the crystal structure is held together by ionic interactions and a number of non‐classical C−H⋅⋅⋅⋅Cl hydrogen bonds, identical to previous data.^[10]^ Table 2 shows the general crystallographic data and details of the refinement of both ILs. It is worth noting that the ratio of Cu and Mn calculated via single‐crystal X‐ray analysis is not as shown by the ICP‐OES measurements. This is due to the fact that here only one crystal from the bulk material is analyzed. Furthermore, the mixed occupancy of the metal ion positions, the very similar electron number, and ion radius of Cu and Mn make it very difficult to clearly distinguish, which electron density belongs to which metal. Additional data of bond lengths, bond angles, and hydrogen bonds are summarized in the Supporting Information (Tables S2 to S9, Figures S2 and S3, Supporting Information).

**Table 2:** Crystallographic data and details of the refinement of **IL13** and **IL2**.

Compounds	[C_4_Py]_2_[MnCl_4_] (**IL13**)	[C_4_Py]_2_[Cu_0.41_Mn_0.59_Cl_4_] (**IL2**)
molecular formula	C36 H56 Cl8 Mn2 N4	C36 H56 Cl8 Cu0.81 Mn1.19 N4
*M* [g mol^−1^]	938.32	945.27
crystal system	monoclinic	monoclinic
space group	*P*2_1_/*n* (14)	*P*2_1_/*n* (14)
*T* [K]	210(2)	293
*a*, *b, c* [Å]	15.4716(7), 18.8829(8), 16.8042(9)	15.6454(6), 18.4549(7), 16.8552(7)
*α*, *β*, *γ* [°]	90, 110.752(4), 90	90, 110.421(3), 90
*V* [Å^3^]	4590.8(4)	4560.8(3)
*Z*	4	4
*D*_calcd_ [g cm^‐3^]	1.358	1.377
*μ* [mm^−1^]	1.045	1.205
reflns collected	79510	80709
independent reflns	8066	4821
*R*int	0.0782	0.0495
no. reflns with *I*>2*σ*(*I*)	8066	8009
no. refined parameters	451	471
*R*1, *wR*2^[a]^ [*I*>2*σ*(*I*)]	0.0332, 0.0517	0.0506, 0.1176
*R*1, *wR*2^[a]^ (all data)	0.0809, 0.0590	0.0936, 0.1360
GooF on *F* ^2^	0.745	0.947
CCDC	2012411	2012412

[a] *w*=1/[*σ* ^2^(*F*_0_^2^)+(0.0166*P*)^2^] where *P*=(*F*_0_^2^+2*F*_c_^2^)/3 for **IL13** and *w*=1/[*σ* ^2^(*F*_0_^2^)+(0.0739*P*)^2^] where *P*=(*F*_0_^2^+2*F*_c_^2^)/3 for **IL2**.

**Figure 4:**
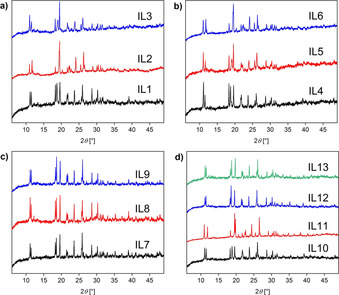
X‐ray diffractograms of a) the copper–manganese ILs (**IL1**–**3**, Table 1), b) the copper–cobalt ILs (**IL4**–**6**, Table 1), c) the cobalt‐manganese‐ILs (**IL7**–**9**, Table 1) and d) monometallic ILs (**IL11–13**) and the trimetallic (**IL10**, Table 1). It must be noted that the Cu‐based IL has already been extensively studied and the current data match the previous information on chemical analysis, crystal structure, and further properties.^[39]^ The *y*‐axis shows the intensity (in a.u.).

The crystal structure of all solid products was further examined using powder X‐ray diffraction (XRD, Figure 4 **)**. XRD proves that all ILs are crystalline at room temperature and the XRD patterns also show that all solids are isostructural. It must however be noted that the patterns are rather noisy and have a background that is not entirely flat. As such, these data indicate that, while the materials crystallize, they likely contain a higher number of defects and possibly some of the solid is not highly ordered. In spite of this, XRD confirms earlier data on the Cu‐based IL (**IL11**)^[10]^ and the XRD data clearly show that exchange of a first row d‐metal cation for another one (or mixing two or three cations) does not lead to drastic changes in the crystal structure.

**Figure 5:**
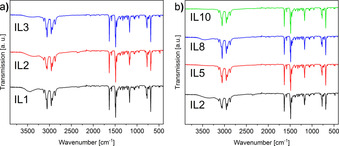
IR spectra of a) the Cu‐Mn‐ILs (**IL1**–**3**) and b) the 50:50 mixtures Cu_0.50_Mn_0.50_‐IL (**IL2**), the Cu_0.50_Co_0.50_‐IL (**IL5**), the Co_0.50_Mn_0.50_‐IL (**IL8**) and of Cu_0.33_Co_0.33_Mn_0.33_‐IL (**IL10**).

Figure 5 shows the corresponding IR‐spectra of the respective samples. The IR spectra of the individual samples are essentially identical. The samples only slightly differ in the water fractions as indicated by the different intensities of the absorption bands at 3500 to 3000 cm^−1^. Besides, all ILs show absorption bands that can be assigned to the aromatic C=C and C=N stretching vibrations of the pyridinium ring at 1630, 1580, and 1490 cm^−1^. The typical O−H deformation vibrations can be found from 1390 to 1210 cm^−1^. The absorption bands at 1170 and 958 cm^−1^ can be assigned to (exocyclic) C−N stretching vibrations, which are characteristic of alkylated pyridinium salts. Furthermore, aromatic deformation vibrations are observed at 770, 730, and 680 cm^−1^. Overall, IR spectroscopy confirms the presence of the pyridinium cation in the solids.

**Figure 6:**
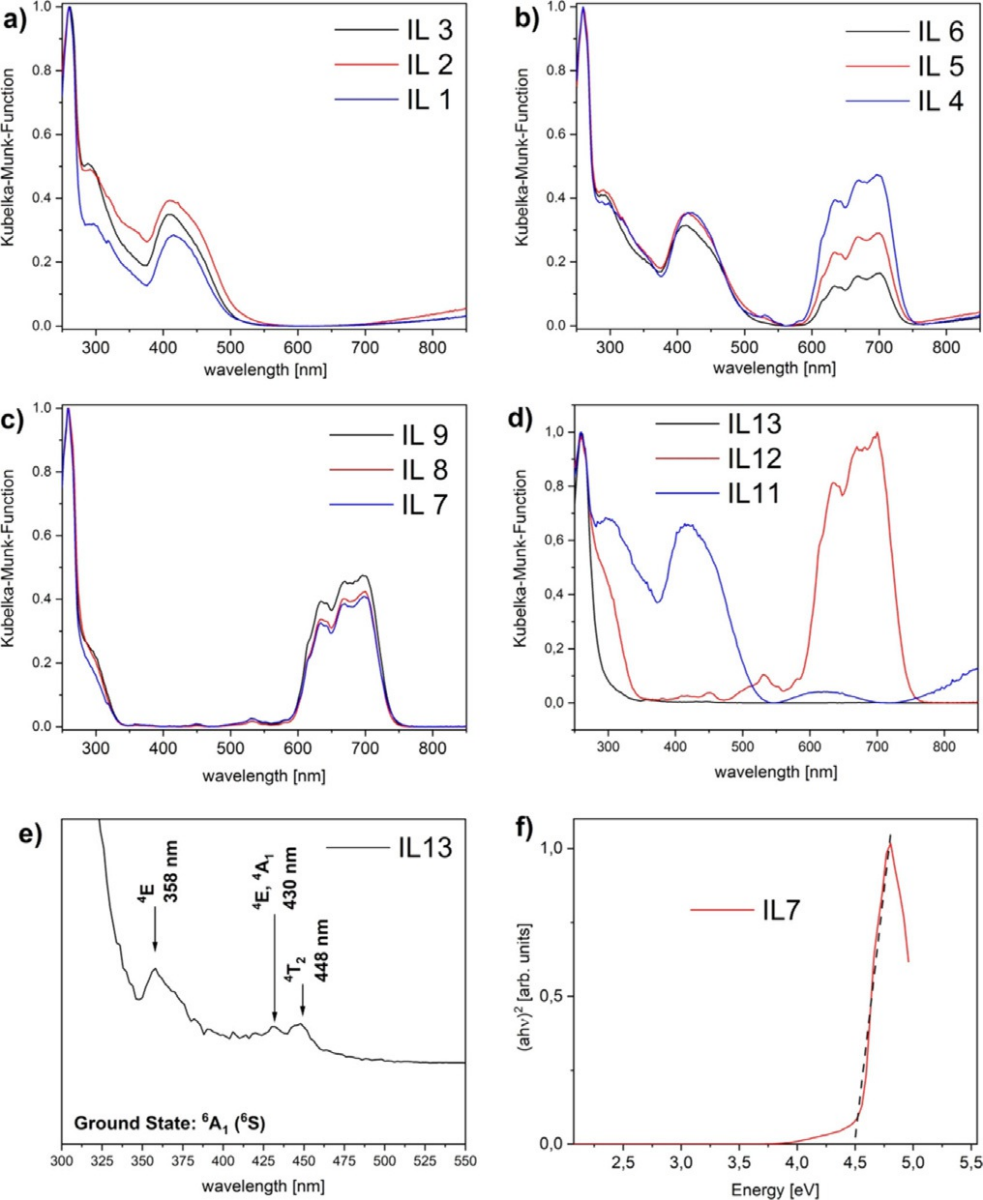
UV/Vis reflectance spectra of a) the Cu–Mn ILs (**IL1**–**3**), b) the Cu–Co ILs (**IL4**–**6**), c) the Co–Mn ILs (**IL7**–**9**) and d) the monometallic ILs (**IL11**–**13**). Figure e) shows a magnified spectrum from 300 to 550 nm of **IL13**. Figure f) shows a representative Tauc‐Plot of **IL7**.

Figure 6 shows representative UV/Vis reflectance data. The Cu–Mn ILs (Figure 6 a) show absorption bands at 290 and 420 nm. In contrast, the Cu–Co compounds (Figure 6 b) show absorption bands at 290, 420, 640, 670, and 690 nm. The bands at 640, 670, and 690 nm can be assigned to the d–d transitions of Co^2+^ in tetrahedral coordination.[^40^, ^41^, ^42^] The absorption band at 420 nm can be assigned to the d–d transitions of Cu^2+^.[^39^, ^43^] The absorption band at 290 nm can be assigned to Cu^2+^.^[44]^ Especially these samples also show a direct relationship between the cobalt content and the absorption band intensity.

Figure 6 c displays the results obtained from the Co‐Mn ILs, which also show absorption bands at 640, 670, and 690 nm. These absorption bands can be assigned to d–d transitions of Co^2+^ in a tetrahedral geometry.[^40^, ^41^, ^42^] The absorption band at around 420 nm can be assigned to either another Co‐absorption band or a possible absorption band of Cu^2+^.[^39^, ^40^, ^41^, ^42^, ^43^] Again a directly proportional relationship between the absorption band intensity and the content of cobalt in the IL can be found.

Figure 6 d shows the results obtained from the monometallic ILs. The copper‐based IL (**IL11**) shows absorption bands at 290 and 420 nm. Both can be assigned to Cu^2+^.[^39^, ^43^, ^44^] The cobalt‐based IL (**IL12**) shows absorption bands at 640, 670, and 690 nm. Those can be assigned to assigned to d–d transitions of Co^2+^ in a tetrahedral geometry.[^40^, ^41^, ^42^] The manganese‐based IL (**IL13**) only shows very weak absorption bands, Figure 6 e, at 358, 430, and 448 nm. Those can be assigned to the spin‐forbidden transitions of Mn^2+^.^[45]^

In addition, UV/Vis spectroscopy was used to determine the direct optical bandgap from the corresponding Tauc plots, Figure 6 f.[^46^, ^47^, ^48^, ^49^, ^50^, ^51^] Table S12 summarizes the results from all ILs. Generally, the direct optical bandgaps indicate that the ILs are large bandgap semiconductors.[^52^, ^53^, ^54^, ^55^]

**Figure 7:**
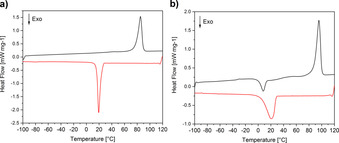
Representative DSC data (second heating (black) and cooling (red) run) of a) **IL5** and b) **IL9**.

Besides the optical properties, we have studied the thermal properties of the ILs. Figure 7 shows representative differential scanning calorimetry (DSC) data and Table S10 summarizes the data. The ILs melt between 69 and 93 °C, which is consistent with existing examples^[10]^ and previous data on **IL11**‐**13**.^[10]^ The ILs have a lower melting point than pure *N*‐butylpyridinium chloride (*T*_m_=160 °C) and the melting peaks occasionally consist of two steps or two combined peaks. Occasionally, glass transition and cold crystallization processes are observed. However, these do not follow a pattern, independent of heating/cooling rates. The crystallization temperature observed upon cooling is between −12 and +32 °C. After repeated heating and cooling a broadening or disappearance of the crystallization peaks is observed; these processes can then be observed as cold crystallizations during the subsequent heating cycle.

Thermogravimetric analysis (TGA) shows that all ILs are thermally stable up to 250 °C and exhibit a weight loss in two steps. The first steps occur at around 250 °C with a weight loss of about 65 to 70 %, while the second step occurs at around 400 °C. Overall a weight loss of 80 to 95 % is observed for all ILs (Supporting Information, Figure S4).

**Figure 8:**
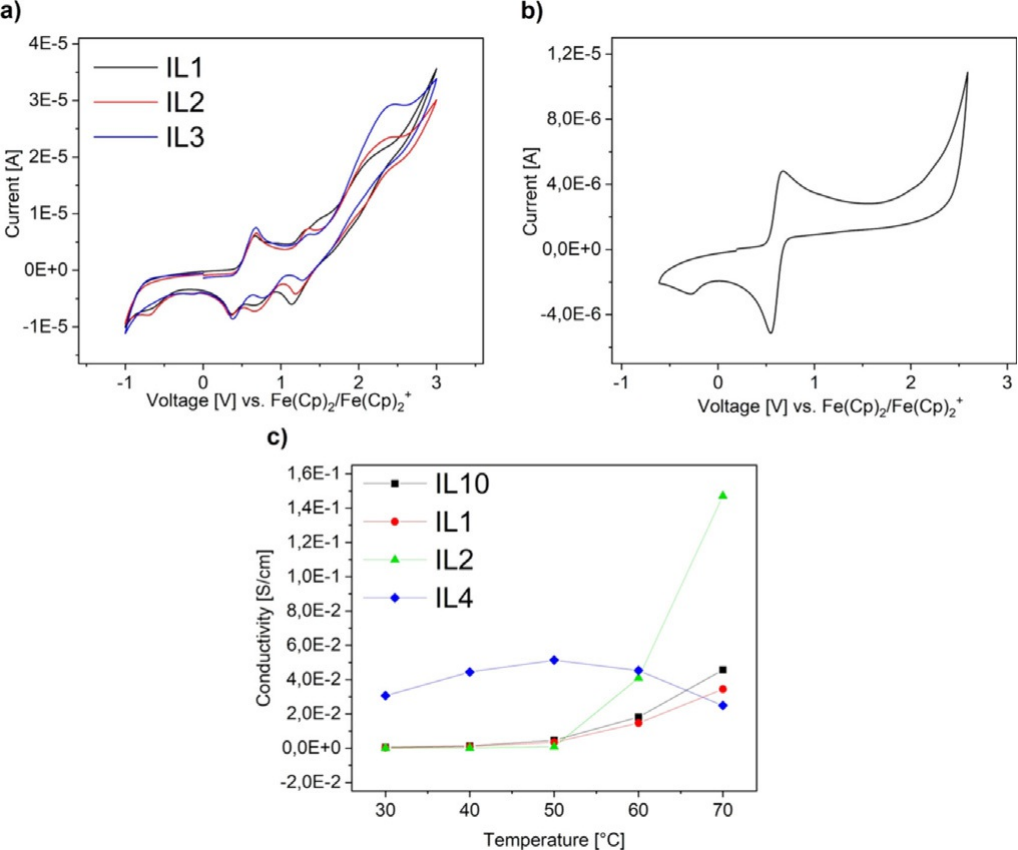
Cyclic voltammograms of a) the Cu‐Mn ILs (**ILs1**–**3**) and b) the ferrocene standard. c) Conductivities of **IL1**, **IL2**, **IL4**, and **IL10** vs. temperature.

Lastly, the electrochemical properties of the ILs were analyzed. The working range was determined via cyclic voltammetry (CV). Here, the working range is equal to the potential range where the ILs are not electrolyzed or decomposed. Simultaneously, CV measurements were also used to identify oxidation and reduction processes in the range of −1–3 V. The ILs were measured in dry acetonitrile. Figure 8 a shows the cyclic voltammograms of the **IL1** to **3** (Cu–Mn‐ILs). To prevent electrochemical degradation the potential limits were set at −1 and +3 V, since the CVs of the ILs show a significant current decrease below −1 V and a notable current increase above 3 V. Furthermore, the majority of the ILs does not show (reversible) redox steps beyond these potential limits. CVs of the Cu–Mn ILs (Figure 8 a) show two oxidation peaks at 0.69 and 1.36 V. During the reduction, four peaks at around −0.78, 0.35, 0.65, and 1.19 V can be observed. These peaks appear in all three Cu–Mn ILs and only slightly differ in the peak location (deviation of ±0.1 V). Similarly, the Co–Mn ILs and the Cu–Co ILs show nearly identical patterns with respect to their reduction and oxidation peaks while only slightly differing in the peak location. The small deviations regarding the peak locations are probably the result of small amounts of water and impurities remaining in the compound.

Due to the fact that the composition of the ILs are known, it is possible to assign the CV signals to specific reduction and oxidation processes. A comparison of the CVs with the CV of ferrocene (Figure 8 b) shows that the peak at 0.67 V can be assigned to ferrocene oxidation to ferrocenium. The second peak at 1.36 V can be assigned to the oxidation of chloride with a standard potential of 1.36 V. The Cu oxidation peak is probably not visible due to overlap with the ferrocene peak. The Mn oxidation peak is not observed due to it being outside the set potential limits of the experiment.

The first reduction peak at −0.78 V in **IL1**–**3** (Figure 8 a) can be assigned to the reduction of small amounts of water, which has a standard electrode potential of −0.8 V.^[56]^ Water is present in the ILs due to their hygroscopic nature. The second reduction peak at 0.37 V can be assigned to the reduction of the Cu^2+^, which has a standard electrode potential of 0.34 V. The third peak at 0.64 V can be assigned to the reduction of ferrocenium with *E*°(Fe(Cp)_2_/Fe(Cp)_2_^+^)=0.60 V. The fourth reduction peak at 1.19 V can either be assigned to the reduction of Mn^2+^ with a standard electrode potential of 1.18 V, or to a possible reduction of oxidized chlorine species, which can form as byproducts during the measurement and have standard electrode potentials in the range of 1.2 V. Again, the copper reduction peak is probably overlapping with the ferrocenium reduction peak (standard electrode potential of 0.52 V. Note that the ferrocene/ferrocenium oxidation/reduction peaks do shift significantly in our study, Supporting Information, Figure S8). Furthermore, while at room temperature a Cu^I^ species is not observed even after multiple CV cycles, there is a significant chance for Cu^I^ formation at higher temperatures.

Interestingly, no clear reduction or oxidation peaks stemming from Co could be found. This is probably again due to the fact that the peaks either overlap with other signals or are outside the set potential limits, since single peaks can be observed in the voltammograms of the monometallic ILs (Figure S8). From the CVs the electrochemical windows can be determined. The data show that the ILs are electrochemically stable between −0.9 V and +2.5 V with most of the redox steps occurring between 0 V and 2 V. This is consistent with published data.^[57]^

Furthermore, the ILs were analyzed using impedance spectroscopy.[^58^, ^59^, ^60^, ^61^, ^62^] Table 3 shows the impedance spectroscopy data and measurement parameters as well as the resulting conductivities. Measurements of **IL11** to **IL13** did not result in processable data. Figure 8 c shows the temperature‐dependent conductivity of **IL1**, **IL2**, **IL4**, and **IL10**. The equivalent circuit diagrams used to interpret the data are shown in Table S11 (ESI). The measurements were conducted from 30 to 70 °C. This is due to two factors: (1) the melting points of the MILs are between 69 and 93 °C and (2) the average working temperature in photovoltaic devices, which present a possible area of application, lies between 50 and 65 °C.^[63]^

**Table 3:** Resistances and conductivities of the ILs obtained from impedance spectroscopy.

MIL	*T* [°C]^[a]^	*R* [Ω]^[b]^	Δ*R* [Ω]^[c]^	*σ* [S cm^−1^]^[d]^	MIL	*T* [°C]^[a]^	*R* [Ω]^[b]^	Δ*R* [Ω]^[c]^	*σ* [S cm^−1^]^[d]^
**IL1**	30	4.57×10^4^	±34	2.48×10^−4^	**IL6**	30	6.96×10^5^	±3989	1.41×10^−5^
	40	1.18×10^4^	±13	9.55×10^−4^		40	1.84×10^5^	±444	5.35×10^−5^
	50	3.23×10^3^	±16	3.50×10^−3^		50	3.57×10^4^	±28	2.75×10^−4^
	60	7.73×10^2^	±2	1.46×10^−2^		60	3.76×10^3^	±6	2.61×10^−3^
	70	3.29×10^2^	±4	3.44×10^−2^		70	2.37×10^2^	±2	4.15×10^−2^
**IL2**	30	4.51×10^5^	±686	2.09×10^−5^	**IL7**	30	5.00×10^4^	±93	1.26×10^−4^
	40	8.55×10^4^	±308	1.10×10^−4^		40	2.27×10^4^	±68	2.76×10^−4^
	50	1.09×10^4^	±24	8.64×10^−4^		50	1.08×10^4^	±22	5.82×10^−4^
	60	2.30×10^2^	±13	4.10×10^−2^		60	5.26×10^3^	±9	1.20×10^−3^
	70	6.41×10^4^	±57	1.47×10^−1^		70	8.50×10^2^	±19	7.39×10^−3^
**IL3**	30	8.20×10^4^	±424	1.53×10^−4^	**IL8**	30	3.14×10^4^	±64	2.66×10^−4^
	40	4.22×10^4^	±130	2.98×10^−4^		40	2.08×10^4^	±61	4.03×10^−4^
	50	4.38×10^4^	±478	2.87×10^−4^		50	1.23×10^4^	±23	6.83×10^−4^
	60	9.58×10^4^	±2145	1.31×10^−4^		60	5.58×10^3^	±9	1.50×10^−3^
	70	1.75×10^5^	±3719	7.19×10^−5^		70	2.24×10^3^	±4	3.73×10^−3^
**IL4**	30	3.52×10^2^	±0.58	3.06×10^−2^	**IL9**	30	2.06×10^8^	±1.55×10^7^	5.23×10^−8^
	40	2.42×10^2^	±0.15	4.44×10^−2^		40	8.39×10^7^	±7.27×10^6^	1.28×10^−7^
	50	2.10×10^2^	±0.27	5.13×10^−2^		50	4.13×10^7^	±1.84×10^6^	2.61×10^−7^
	60	2.38×10^2^	±0.13	4.53×10^−2^		60	1.30×10^7^	±2.09×10^5^	8.29×10^−7^
	70	4.33×10^2^	±2.17	2.49×10^−2^		70	2.28×10^6^	±7.39×10^3^	4.72×10^−6^
**IL5**	30	7.35×10^5^	±2030	1.03×10^−5^	**IL10**	30	2.12×10^4^	±56	4.63×10^−4^
	40	1.64×10^5^	±904	4.60×10^−5^		40	7.24×10^3^	±22	1.36×10^−3^
	50	3.73×10^4^	±74	2.02×10^−4^		50	2.12×10^3^	±2	4.64×10^−3^
	60	8.96×10^3^	±20	8.42×10^−4^		60	5.41×10^2^	±1	1.82×10^−2^
	70	2.50×10^3^	±6	3.01×10^−3^		70	2.15×10^2^	±4	4.57×10^−2^

[a] Temperature. [b] Resistance of the bulk phase. [c] Resistance error. [d] Conductivity. Note: Measurements of **IL11**‐**13** did not result in analyzable data due to a rather large scattering of the raw data points.

Temperature‐dependent conductivity measurements show that the conductivity (combined ionic and electronic conductivity) of all ILs ranges between 10^−4^ and 10^−8^ S cm^−1^ near room temperature with **IL4** being the exception at 10^−2^ S cm^−1^. At higher temperatures the conductivities change significantly. At 70 °C they range from 10^−1^ to 10^−3^ S cm^−1^ with **IL3** and **IL9** being the exceptions at 10^−5^ and 10^−6^ S cm^−1^, respectively. Temperature‐dependent data show two general trends. The most common one is a direct correlation between increasing temperature and increasing conductivity. This behavior is shown in Figure 8 c for **IL2** and **IL10**, respectively. This behavior is expected because increasing temperature lead to increasing ion mobilities in the ILs and hence to a higher conductivity. The second trend is illustrated by **IL4** (Figure 8 c). Here the conductivity increases up to 50 °C before decreasing again with increasing temperature.

Overall, the Cu–Mn IL with a ratio of 50:50 (**IL2**) and the trimetallic (**IL10)** show the highest conductivities without any of the detrimental effects such as deposition of foreign mineral phases or electrode corrosion (see Discussion section).

## Discussion

The current article presents new findings on metal‐containing ILs with a focus on the structure and properties of ILs containing two or three different 3d‐metals.

X‐ray diffraction (Figures 2, 3, 4, Tables S2 to S9) shows that all ILs are isostructural and only marginally differ in the unit cell. This is consistent with existing data on similar systems[^10^, ^27^, ^28^, ^35^, ^36^] and confirms that both the pyridinium and the tetrahalidometallate moieties are stable and reliable building blocks for metal‐containing ILs. The first new aspect presented here therefore is the proof that ILs containing two or three 3d‐metals are structural analogues to the respective monometallic ILs.[^10^, ^27^, ^28^, ^29^, ^35^, ^36^]

Consistent with the above, DSC (Figure 7) matches observations on the simpler, monometallic ILs.^[10]^ **IL11** does not show a clean melting or crystallization point, which is probably due to orientation processes and the system reorientation too slow during the measurement. The ILs show a clear melting transition and occasionally a *T*_g_ or a cold crystallization. There is a strong dependence of the melting point from the composition, that is, type(s) of metal in the ILs. Most notably, the melting points of Cu‐based ILs are lower than when no Cu is present in the IL. This effect may be caused by Jahn–Teller distortions around the metal ions induced by the presence of the Cu^II^ d^9^ system.[^10^, ^64^, ^65^, ^66^] Another possible explanation may be that the low melting points are a consequence of the low lattice energy caused by weak anion–π interactions, or higher Coulomb interactions caused by packing effects.

The rather broad signals and the fact that sometimes two peaks are observed in the DSC data (Figures S5–S7) further indicate that, although the compounds crystallize, there is a chance of lower order or possibly even some enrichment of one metal in certain regions of the solid (clustering). Lower order could again be caused in the Cu‐containing ILs due to Jahn–Teller distortion. Similarly, the fact that sometimes cold crystallizations are observed, indicates rather slow crystallization dynamics of the systems. This phenomenon is well known for strongly interacting systems like ILs.^[67]^ This also correlates with the results of the XRD data (Figure 4), where the relatively noisy background indicates that the samples do not crystallize perfectly.

The second new aspect is the fact that this article for the first time describes the optical bandgaps of these intensely colored compounds (Figure 1, Table 3).

Moreover, the bandgaps of the Cu–Mn ILs and the Cu–Co ILs are slightly lower than the bandgaps of the Co‐Mn ILs. As there is a direct connection between direct optical bandgap and electronic conductivity,[^46^, ^51^, ^68^, ^69^] the Cu‐containing ILs (**ILs1**–**6**) may show a somewhat higher electronic conductivity. In the current study, however, this effect overlaps with another set of processes.

As a third new aspect the current study provides the first detailed report on conductivities of tetrachloridometallate(II) ILs. In most cases, the IL conductivity increases with increasing temperature (Figure 8). This behavior is expected due to the higher ionic mobilities in the MILs as they approach the melting point. Overall, the conductivities are comparable to those of other pyridinium‐based ILs and eutectic mixtures.^[70]^ Indeed, the conductivities of the current compounds are slightly higher than comparable compounds at elevated temperatures.^[71]^ Consequently, the current data suggest that the ILs studied here could be interesting electrolytes for intermediate temperature applications such as metal halide batteries.^[72]^ Furthermore, due to the ILs being solid at room temperature they could also be promising electrolytes in solid state electrolyte (SE) batteries.[^73^, ^74^]

Moreover, Cu‐containing ILs show a rather peculiar conductivity vs. temperature (Figure 8). There may be a number of competing processes that lead to this behavior. For one, Cu^2+^ may be reduced to Cu^+^ and Cu^0^ during the measurement due to its relatively high standard electrode potential [*E*°(Cu^2+^/Cu)=+0.35 V; *E*°(Cu^2+^/Cu^+^)=+0.16 V; *E*°(Cu^+^/Cu)= +0.52 V]. Clearly the formation of Cu^0^ species will lead to drastic changes in the conductivities and electrochemical behavior. Moreover, the formation of Cu^+^ will lead to the deposition of CuCl in the presence of chloride anions.[^22^, ^75^] As CuCl is a semiconductor with a bulk bandgap of ca. 3.25 eV,^[75]^ this will again affect the conductivity of the entire system. The assumption of CuCl or Cu formation is also supported by the optical appearance of the compound: the (initially orange) **IL3**, which contains a high amount of Cu, shows green discolorations at its surface, which could be CuCl, after the impedance measurement. This is shown in Figure S9. The high conductivity values may partially be a consequence of the decomposition of the IL and the formation of CuCl, which is known for its high ionic conductivity (4.2×10^−2^ S cm^−1^).[^76^, ^77^, ^78^, ^79^] While this may be the case for **ILs 1**–**3**, some of the other compounds (e.g. **IL7**), which do not contain copper and do not show any evidence of decomposition, also display very high conductivity values of ca. 10^−2^ S cm^−1^.

Furthermore, corrosion also affects the conductivity of the ILs. Corrosive behavior is well‐known for MILs.[^57^, ^80^, ^81^] Indeed, further SEM and EDX analysis of the electrodes shows metal deposition (Figure S10). For example, **IL4**, which shows the best conductivity near room temperature, contains a high amount of cobalt with a small amount of copper. As the experiments were made with gold‐plated nickel electrodes, a reaction between Co^2+^ or Cu^2+^ is highly likely, especially considering the fact that chloride is able to corrode the gold plating (this is confirmed by SEM/EDX data and by the fact that after the experiment, the sample chamber smells of chlorine). As result, Cu and Co deposition may drastically alter the conductivities. Metallic Co shows good ionic conductivity and metallic Cu shows a good electronic conductivity.[^82^, ^83^] Thus Cu metal deposition should in fact lead to an enhanced conductivity even if the Cu deposited are below the percolation threshold.

Considering the question why this unexpected trend in the conductivities is only observed in the Cu‐based ILs, we currently hypothesize that Mn^2+^ may be a key component. Mn^2+^ may act as a redox stabilizer (electron scavenger) preventing the reduction of Cu^2+^ to Cu^+^ or Cu^0^ in all ILs containing at least 25 % of Mn^2+^. Clearly, preventing the formation of semiconducting or even metallic deposits will help maintain the system intact and prevent such an unusual conductivity change.

The current article therefore shows that the combination of several d‐block metals into one single IL with a well‐defined composition produces interesting compounds with tunable properties.

## Conclusions

Although tetrahalidometallate ILs have been studied for a wide range of research and application fields, there is virtually no information on the effects of metal combination in ILs. This article therefore provides new insights into the optical and electrochemical behavior of ILs containing two or even three d‐block metals. The data clearly show that combining metals in ILs can add value to these interesting compounds. In the current examples, the optical bandgaps, the melting transitions, the electrochemical properties, and electronic conductivities can directly be controlled by proper choice of the constituting elements of the ILs. As a result, the current study provides the first prototypes of tunable materials with application potential in batteries, electrolytes, optical elements, solar cells, or light absorbers. Overall, the materials presented here are a highly flexible and adaptable platform for materials development for many different uses.

## Experimental Section

**Chemicals**: *N*‐butylpyridinium chloride (Merck≥98.0 %), CoCl_2_⋅6 H_2_O (VWR≥98.0 %), CuCl_2_⋅2 H_2_O (Fluka Analytical≥99 %), MnCl_2_⋅H_2_O (Carl Roth≥99 %), propan‐2‐ol (VWR ≥99 %), hydrochloric acid (VWR 35 %), magnesium sulfate AnalaR NORMAPUR (VWR≥98.0 %, CAS 7587‐889), acetonitrile (VWR≥99.9 %), tetrabutylammonium hexafluorophosphate (TBAHFP; Alfa Aesar 98 %), and ferrocene (Alfa Aesar 99 %) were used as received.

**Synthesis**: Synthesis was done according to ref.[39] The synthetic procedure was as follows: 200 mg (1.17 mmol) *N*‐butylpyridinium chloride (BuPyCl) and an equimolar amount of the metal salts in the ratio of 2:1 (0.59 mmol in total) were dissolved in 10 mL of 2‐propanol and 0.35 mL HCl (37 %) was added to the solution. The reaction was held at 110 °C for 60 min under reflux. Solvent and additional water were removed by rotary evaporation and the resulting compounds were dried under vacuum (10^−3^ bar) for 12 h. The resulting compounds were directly used for further analysis without additional purification. For detailed mass calculation and yield see Table S13. Generally, the yields of the ILs are between 90 and 97 %, with **IL5** (83 %) and **IL13** (78 %) being the exceptions. The synthesis was repeated up to five times for reproducibility. The crystallization of the ILs were done as follows: a few amounts of the ILs were dissolved in various solvents, for example, 2‐propanol, acetonitrile or methanol. After several days or weeks crystals were formed under solvent evaporation and used for X‐ray crystal structure analysis.

**Infrared spectroscopy**: IR measurements were conducted using a NICOLET iS5 by Thermo Scientific with an ID7 ATR‐attachment with diamond crystal. Samples were measured as powder in ATR mode between 400 and 4000 cm^−1^ with a resolution of 4 cm^−1^.

**Thermogravimetric analysis**: TGA measurements were conducted using a TGA 4000 Thermal Analyzer by PerkinElmer at a heating rate of 10 K min^−1^ under static air atmosphere.

**Crystal structure analysis**: For details, see the Supporting Information. Deposition numbers 2012411, and 2012412 (**IL13**, and **IL2**) contain the supplementary crystallographic data for this paper. These data are provided free of charge by the joint Cambridge Crystallographic Data Centre and Fachinformationszentrum Karlsruhe Access Structures service.

Data collection was performed with a STOE StadiVari diffractometer (four‐circle goniometer) with a Genix Microfocus X‐ray source (Mo‐Kα‐radiation, *λ*=0.71073 Å) and a Dectris 200 K detector. The data were corrected for absorption as well as for Lorentz and polarization effects using the program X‐Area.^[84]^ The structures were solved using SHELXS‐2013/2,^[85]^ refined by the program SHELXL‐2014/7^[86]^ and the crystal structures were visualized with Diamond 4.^[87]^

**Powder X‐ray diffraction**: PXRD date were collected using a PANalytical Empyrean powder X‐ray diffractometer operating at 40 kV and 40 mA. The diffractometer was configured with a focusing X‐ray mirror for Cu radiation (*λ*=1.5419 Å) and a PIXcel1D detector. Scans were run for 61 min over a 2*θ* range of 4–70° with a step size of 0.0131°.

**Differential scanning calorimetry**: DSC measurements were done on a Netzsch DSC 214 Polyma at 10 K min^−1^ under nitrogen. Each run consisted of three heating‐cooling cycles.

**Elemental analysis**: CHN analysis was carried out using an Elementar vario EL III analyzer. Limit of detection is 0.3 %.

**Electrochemistry**: Cyclic voltammetry (CV) measurements were performed using the three‐electrode system of a Metrohm Autolab PGSTAT204. For CV measurements the ILs (0.1 m in dry acetonitrile) were placed between a Pt counter electrode with a diameter of 1 cm^2^ (with a central hole for reference electrode) as well as a glassy carbon working electrode. A silver electrode with a diameter of 2 mm was used as the reference electrode. As a reference ferrocene (5×10^−4^ m) was used. For the pure ferrocene CV measurement tetrabutylammonium hexafluorophosphate (0.1 m) was used as a conductive agent.

Impedance spectra were measured using the impedance setup of the Autolab PGSTAT204 system (Metrohm GmbH & Co. KG) from 10^5^ Hz to 1 Hz with an amplitude of 0.1 V. For the temperature dependent impedance measurements a temperature‐controlled microcell HC (rhd instruments) was used and carried out at temperatures ranging from 25 °C to 70 °C. Heating and cooling was done by means of a Peltier element with an accuracy of 0.01 °C. The compounds were prepared as follows: Each IL was placed in a silicon form and held at 110 °C in a heating oven for 1 h. This process produces a macroscopic pellet after cooling and also removes residual water. The pellets were then placed in the measurement cell between the working electrodes. The conductivity *σ* was calculated using Equation 1 with *d* being the height of the sample, *R* being the resistance and *A* being the surface content of the circular sample.

(1) σ=d/(R×A)

**Electrode and cell assembly**: The performance of the pure ILs was characterized in a two‐electrode cell configuration (Pt‐electrode, Graphit‐electrode) with a rhd TSC battery (Ni electrodes, diameter 9 mm) in combination with a Metrohm Autolab PGSTAT204. The Ni electrodes were later replaced by Au electrodes due to the presence of corrosive processes. For the analysis of the generated impedance results, the RelaxIS software (rhd, instruments, Marburg, Germany) was used.

**Inductively coupled plasma optical emission spectroscopy**: ICP OES measurements were performed using the PerkinElmer Optical Emission Spectrometer Optima 5300 DV with the Scott‐Chamber/Cross‐Flow‐Nebulizer. Read time was 2–10 s with a power of 1400 W and plasma gas flow of 17 L min^−1^, auxiliary gas flow of 0.2 L min^−1^ and nebulizer gas flow of 0.6 L min^−1^. The measurement was performed axially.

**UV/Vis‐spectroscopy**: UV/Vis measurements were conducted using a Lambda 950 by PerkinElmer with the solid material attachment Praying MantisTM by Harrick Scientific Products INC. During the experiments MgSO_4_ AnalaR NORMAPUR by VWR was used as a background material. The measuring range was *λ*=850‐250 nm with a resolution of 2 nm. The Kubelka–Munk Equation 2 was used for the analysis of the UV/Vis data.

(2) $$\frac{K}{S}=\frac{{\left(1-R\right)}^{2}}{2R}$$

*K=*absorption coefficient, *S*=scattering coefficient, *R*=reflectance

Based on the UV/Vis analysis the optical bandgaps were graphically analyzed using the Tauc plot (3). The plots were then fitted using Origin [Eq. 3].

(3) $${\left(\alpha hv\right)}^{1/n}=A\left(hv-E_{g}\right)$$

*h*=Planck's constant, *ν*=photon's frequency, *α*=absorption coefficient, *E*_g_=bandgap, *A*=proportionality constant with the following values for n: *n*=1/2 for direct allowed transitions, *n*=3/2 for direct forbidden transitions, *n*=2 for indirect allowed transitions, *n*=3 for indirect forbidden transitions.

## Conflict of interest

The authors declare no conflict of interest.

## Supporting information

As a service to our authors and readers, this journal provides supporting information supplied by the authors. Such materials are peer reviewed and may be re‐organized for online delivery, but are not copy‐edited or typeset. Technical support issues arising from supporting information (other than missing files) should be addressed to the authors.

Supplementary
