# Supplementary material for: Ionic Liquids with More than One Metal: Optical and Electrochemical Properties versus d‐Block Metal Combinations
Source: Chemistry. 2020 Dec 3;26(72):17504–13. doi: 10.1002/chem.202003097 (PMC7839689; doi:10.1002/chem.202003097)
Supplement: Supplementary file 1 — Supplementary [file CHEM-26-17504-s001.pdf]

Chemistry–A European Journal

Supporting Information

Ionic Liquids with More than One Metal: Optical and Electrochemical Properties versus d-Block Metal Combinations

Christian Balischewski,[a] Karsten Behrens,[a] Kerstin Zehbe,[a] Christina Günter,[b] Stefan Mies,[a] Eric Sperlich,[a] Alexandra Kelling,[a] and Andreas Taubert*[a]

Author Contributions

C.B. Data curation: Equal; Formal analysis: Lead; Investigation: Equal; Methodology: Equal; Writing - Original Draft: Lead; Writing - Review & Editing: Equal
K.B. Data curation: Equal; Formal analysis: Equal; Investigation: Equal; Methodology: Equal; Writing - Original Draft: Equal
K.Z. Data curation: Equal; Formal analysis: Equal; Investigation: Equal; Validation: Equal; Writing - Review & Editing: Equal
C.G. Data curation: Supporting; Formal analysis: Equal; Methodology: Supporting; Validation: Equal; Writing - Review & Editing: Supporting
S.M. Data curation: Supporting; Formal analysis: Supporting; Investigation: Supporting; Methodology: Equal; Software: Equal; Validation: Supporting; Writing - Original Draft: Supporting; Writing - Review & Editing: Supporting
E.S. Data curation: Equal; Formal analysis: Equal; Investigation: Equal; Writing - Original Draft: Supporting; Writing - Review & Editing: Supporting
A.T. Data curation: Equal; Formal analysis: Equal; Funding acquisition: Lead; Investigation: Lead; Project administration: Lead; Supervision: Lead; Writing - Original Draft: Equal; Writing - Review & Editing: Lead
A.K. Data curation: Supporting; Formal analysis: Supporting; Investigation: Supporting; Methodology: Supporting; Software: Supporting; Validation: Supporting; Visualization: Supporting; Writing - Original Draft: Supporting.

Table of Contents

1. ICP OES 2
2. Crystal structures 3
3. TG and DSC measurements 9
4. CV- and Impedance measurements 13
5. SEM and EDX measurements 17
6. UV/Vis measurements 18
7. General Synthesis 18

1. ICP OES

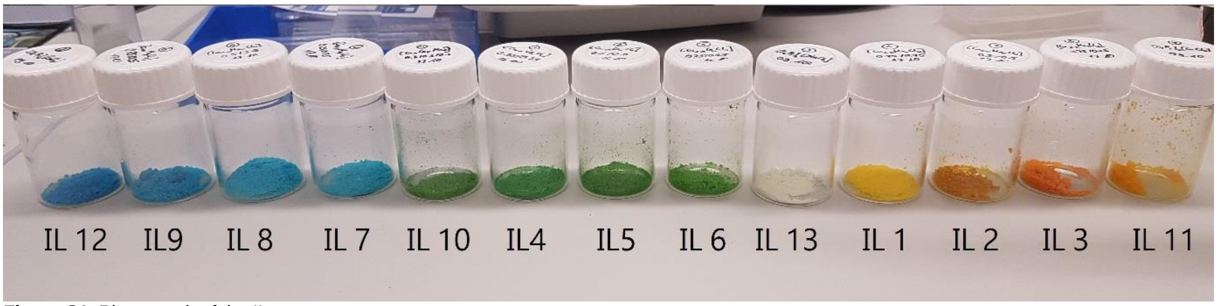

Figure S1. Photograph of the ILs.

Table S1. Elemental analysis data of the ILs.

Compound	Abbreviation		C [w%]	H [w%]	N [w%]
$[C_4Py]_2[Cu_{0.50}Mn_{0.50}Cl_4]$	IL 2	Calc.	45.66	5.96	5.92
		Meas.	44.10 ± 0.15	7.45 ± 0.12	5.722 ± 0.04
$[C_4Py]_2[Cu_{0.50}Co_{0.50}Cl_4]$	IL 5	Calc.	45.89	5.99	5.95
		Meas.	44.67 ± 0.14	6.55 ± 0.01	5.79 ± 0.01
$[C_4Py]_2[Co_{0.50}Mn_{0.50}Cl_4]$	IL 8	Calc.	45.73	5.97	5.95
		Meas.	44.94 ± 0.01	6.98 ± 0.03	5.84 ± 0.02
$[C_4Py]_2[Cu_{0.33}Co_{0.33}Mn_{0.33}Cl_4]$	IL 10	Calc.	45.73	5.97	5.95
		Meas.	44.88 ± 0.16	6.52 ± 0.05	5.80 ± 0.01

2. Crystal structures

$(C_4Py)_2[MnCl_4]$ (IL 13)

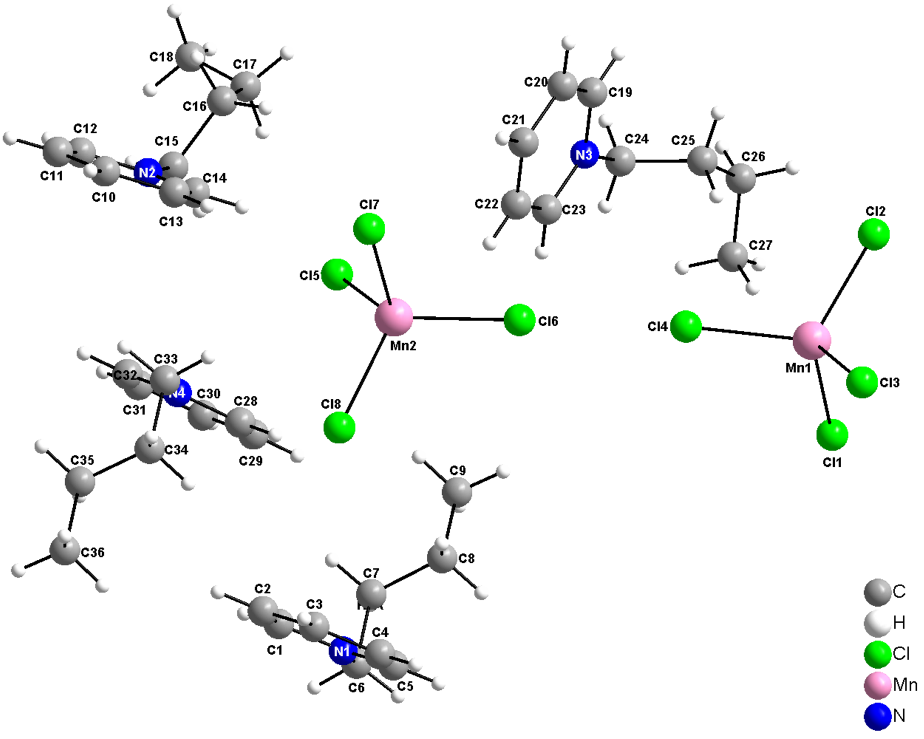

Figure S 2. Asymmetric unit of IL 13. For better overview the hydrogen labels are excluded.

Table S2. Bond lengths [Å] of **IL 13**.

Bond	d [Å]	Bond	d [Å]	Bond	d [Å]
C1—N1	1.341 (4)	C15—H15B	0.9800	C29—H29	0.9400
C1—C2	1.373 (4)	C16—C17	1.520 (5)	C30—C31	1.376 (5)
C1—H1	0.9400	C16—H16A	0.9800	C30—H30	0.9400
C2—C3	1.372 (5)	C16—H16B	0.9800	C31—C32	1.369 (5)
C2—H2	0.9400	C17—C18	1.434 (6)	C31—H31	0.9400
C3—C4	1.375 (6)	C17—H17A	0.9800	C32—N4	1.335 (4)
C3—H3	0.9400	C17—H17B	0.9800	C32—H32	0.9400
C4—C5	1.369 (5)	C18—H18A	0.9700	C33—N4	1.489 (4)
C4—H4	0.9400	C18—H18B	0.9700	C33—C34	1.508 (5)
C5—N1	1.333 (4)	C18—H18C	0.9700	C33—H33A	0.9800
C5—H5	0.9400	C19—N3	1.348 (4)	C33—H33B	0.9800
C6—N1	1.488 (4)	C19—C20	1.358 (5)	C34—C35	1.462 (5)
C6—C7	1.514 (5)	C19—H19	0.9400	C34—H34A	0.9800
C6—H6A	0.9800	C20—C21	1.380 (5)	C34—H34B	0.9800
C6—H6B	0.9800	C20—H20	0.9400	C31—H31	0.9400
C7—C8	1.508 (4)	C21—C22	1.371 (5)	C32—N4	1.335 (4)
C7—H7A	0.9800	C21—H21	0.9400	C32—H32	0.9400
C7—H7B	0.9800	C22—C23	1.361 (5)	C33—N4	1.489 (4)
C8—C9	1.507 (5)	C22—H22	0.9400	C33—C34	1.508 (5)
C8—H8A	0.9800	C23—N3	1.339 (4)	C33—H33A	0.9800
C8—H8B	0.9800	C23—H23	0.9400	C33—H33B	0.9800
C9—H9A	0.9700	C24—N3	1.488 (4)	C34—C35	1.462 (5)
C9—H9B	0.9700	C24—C25	1.524 (4)	C34—H34A	0.9800
C9—H9C	0.9700	C24—H24A	0.9800	C34—H34B	0.9800
C10—N2	1.335 (4)	C24—H24B	0.9800	C35—C36	1.568 (6)
C10—C11	1.374 (4)	C25—C26	1.517 (5)	C35—H35A	0.9800
C10—H10	0.9400	C25—H25A	0.9800	C35—H35B	0.9800
C11—C12	1.371 (5)	C25—H25B	0.9800	C36—H36A	0.9700
C11—H11	0.9400	C26—C27	1.515 (5)	C36—H36B	0.9700
C12—C13	1.383 (5)	C26—H26A	0.9800	C36—H36C	0.9700
C12—H12	0.9400	C26—H26B	0.9800	Cl1—Mn1	2.3808 (10)
C13—C14	1.360 (4)	C27—H27A	0.9700	Cl2—Mn1	2.3515 (10)
C13—H13	0.9400	C27—H27B	0.9700	Cl3—Mn1	2.3795 (10)
C14—N2	1.348 (4)	C27—H27C	0.9700	Cl4—Mn1	2.3547 (9)
C14—H14	0.9400	C28—N4	1.344 (4)	Cl5—Mn2	2.3826 (10)
C15—N2	1.498 (4)	C28—C29	1.367 (5)	Cl6—Mn2	2.3502 (9)
C15—C16	1.528 (5)	C28—H28	0.9400	Cl7—Mn2	2.3783 (10)
C15—H15A	0.9800	C29—C30	1.369 (5)	Cl8—Mn2	2.3572 (10)

Table S3. Bond angles [°] of **IL 13**.

Bond angle	∠ [°]	Bond angle	∠ [°]	Bond angle	∠ [°]
N1—C1—C2	120.8 (3)	C17—C16—H16A	108.7	C30—C29—H29	120.2
N1—C1—H1	119.6	C15—C16—H16A	108.7	C29—C30—C31	119.5 (4)
C2—C1—H1	119.6	C17—C16—H16B	108.7	C29—C30—H30	120.2
C3—C2—C1	119.1 (4)	C15—C16—H16B	108.7	C31—C30—H30	120.2
C3—C2—H2	120.5	H16A—C16—H16B	107.6	C32—C31—C30	118.9 (3)
C1—C2—H2	120.5	C18—C17—C16	114.5 (4)	C32—C31—H31	120.6
C2—C3—C4	119.4 (4)	C18—C17—H17A	108.6	C30—C31—H31	120.6
C2—C3—H3	120.3	C16—C17—H17A	108.6	N4—C32—C31	121.1 (3)
C4—C3—H3	120.3	C18—C17—H17B	108.6	N4—C32—H32	119.5
C5—C4—C3	119.4 (4)	C16—C17—H17B	108.6	C31—C32—H32	119.5
C5—C4—H4	120.3	H17A—C17—H17B	107.6	N4—C33—C34	113.8 (3)
C3—C4—H4	120.3	C17—C18—H18A	109.5	N4—C33—H33A	108.8
N1—C5—C4	120.8 (3)	C17—C18—H18B	109.5	C34—C33—H33A	108.8
N1—C5—H5	119.6	H18A—C18—H18B	109.5	N4—C33—H33B	108.8
C4—C5—H5	119.6	C17—C18—H18C	109.5	C34—C33—H33B	108.8
N1—C6—C7	112.3 (2)	H18A—C18—H18C	109.5	H33A—C33—H33B	107.7
N1—C6—H6A	109.1	H18B—C18—H18C	109.5	C35—C34—C33	114.1 (3)
C7—C6—H6A	109.1	N3—C19—C20	120.6 (3)	C35—C34—H34A	108.7
N1—C6—H6B	109.1	N3—C19—H19	119.7	C33—C34—H34A	108.7
C7—C6—H6B	109.1	C20—C19—H19	119.7	C35—C34—H34B	108.7
H6A—C6—H6B	107.9	C19—C20—C21	119.9 (3)	C33—C34—H34B	108.7
C8—C7—C6	115.2 (3)	C19—C20—H20	120.1	H34A—C34—H34B	107.6
C8—C7—H7A	108.5	C21—C20—H20	120.1	C34—C35—C36	112.1 (3)
C6—C7—H7A	108.5	C22—C21—C20	118.6 (3)	C34—C35—H35A	109.2
C8—C7—H7B	108.5	C22—C21—H21	120.7	C36—C35—H35A	109.2
C6—C7—H7B	108.5	C20—C21—H21	120.7	C34—C35—H35B	109.2
H7A—C7—H7B	107.5	C23—C22—C21	120.1 (3)	C36—C35—H35B	109.2
C9—C8—C7	113.4 (3)	C23—C22—H22	120.0	H35A—C35—H35B	107.9
C9—C8—H8A	108.9	C21—C22—H22	120.0	C35—C36—H36A	109.5
C7—C8—H8A	108.9	N3—C23—C22	120.7 (3)	C35—C36—H36B	109.5
C9—C8—H8B	108.9	N3—C23—H23	119.6	H36A—C36—H36B	109.5
C7—C8—H8B	108.9	C22—C23—H23	119.6	C35—C36—H36C	109.5
H8A—C8—H8B	107.7	N3—C24—C25	111.1 (2)	H36A—C36—H36C	109.5
C8—C9—H9A	109.5	N3—C24—H24A	109.4	H36B—C36—H36C	109.5
C8—C9—H9B	109.5	C25—C24—H24A	109.4	Cl2—Mn1—Cl4	114.53 (4)
H9A—C9—H9B	109.5	N3—C24—H24B	109.4	Cl2—Mn1—Cl3	107.06 (3)
C8—C9—H9C	109.5	C25—C24—H24B	109.4	Cl4—Mn1—Cl3	109.99 (4)
H9A—C9—H9C	109.5	H24A—C24—H24B	108.0	Cl2—Mn1—Cl1	111.85 (4)
H9B—C9—H9C	109.5	C26—C25—C24	112.8 (2)	Cl4—Mn1—Cl1	107.44 (3)
N2—C10—C11	120.4 (3)	C26—C25—H25A	109.0	Cl3—Mn1—Cl1	105.62 (4)
N2—C10—H10	119.8	C24—C25—H25A	109.0	Cl6—Mn2—Cl8	116.32 (4)
C11—C10—H10	119.8	C26—C25—H25B	109.0	Cl6—Mn2—Cl7	107.88 (3)
C12—C11—C10	119.2 (3)	C24—C25—H25B	109.0	Cl8—Mn2—Cl7	109.46 (4)
C12—C11—H11	120.4	H25A—C25—H25B	107.8	Cl6—Mn2—Cl5	108.90 (4)
C10—C11—H11	120.4	C27—C26—C25	113.9 (3)	Cl8—Mn2—Cl5	108.29 (3)
C11—C12—C13	119.4 (3)	C27—C26—H26A	108.8	Cl7—Mn2—Cl5	105.46 (4)
C11—C12—H12	120.3	C25—C26—H26A	108.8	C5—N1—C1	120.5 (3)
C13—C12—H12	120.3	C27—C26—H26B	108.8	C5—N1—C6	121.4 (3)
C14—C13—C12	119.7 (3)	C25—C26—H26B	108.8	C1—N1—C6	118.1 (3)
C14—C13—H13	120.2	H26A—C26—H26B	107.7	C10—N2—C14	121.3 (3)
C12—C13—H13	120.2	C26—C27—H27A	109.5	C10—N2—C15	119.3 (3)
N2—C14—C13	120.0 (3)	C26—C27—H27B	109.5	C14—N2—C15	119.3 (3)
N2—C14—H14	120.0	H27A—C27—H27B	109.5	C23—N3—C19	120.2 (3)
C13—C14—H14	120.0	C26—C27—H27C	109.5	C23—N3—C24	120.4 (3)
N2—C15—C16	109.7 (2)	H27A—C27—H27C	109.5	C19—N3—C24	119.4 (3)
N2—C15—H15A	109.7	H27B—C27—H27C	109.5	C32—N4—C28	120.5 (3)
C16—C15—H15A	109.7	N4—C28—C29	120.4 (3)	C32—N4—C33	120.9 (3)
N2—C15—H15B	109.7	N4—C28—H28	119.8	C28—N4—C33	118.6 (3)
C16—C15—H15B	109.7	C29—C28—H28	119.8		
H15A—C15—H15B	108.2	C28—C29—C30	119.6 (4)		
C17—C16—C15	114.1 (3)	C28—C29—H29	120.2		

Table S4. Torsion angles [°] of **IL 13**.

Torsion angle	∠ [°]	Torsion angle	∠ [°]
N1—C1—C2—C3	-0.3 (5)	C4—C5—N1—C1	1.0 (5)
C1—C2—C3—C4	0.5 (6)	C4—C5—N1—C6	-179.1 (3)
C2—C3—C4—C5	0.0 (6)	C2—C1—N1—C5	-0.5 (5)
C3—C4—C5—N1	-0.7 (6)	C2—C1—N1—C6	179.7 (3)
N1—C6—C7—C8	68.9 (4)	C7—C6—N1—C5	-103.9 (4)
C6—C7—C8—C9	175.9 (3)	C7—C6—N1—C1	76.0 (3)
N2—C10—C11—C12	0.5 (5)	C11—C10—N2—C14	0.3 (5)
C10—C11—C12—C13	-0.8 (5)	C11—C10—N2—C15	-175.3 (3)
C11—C12—C13—C14	0.4 (5)	C13—C14—N2—C10	-0.7 (5)
C12—C13—C14—N2	0.4 (5)	C13—C14—N2—C15	174.8 (3)
N2—C15—C16—C17	-174.8 (3)	C16—C15—N2—C10	103.5 (3)
C15—C16—C17—C18	74.3 (5)	C16—C15—N2—C14	-72.1 (4)
N3—C19—C20—C21	-0.8 (5)	C22—C23—N3—C19	-0.4 (5)
C19—C20—C21—C22	0.4 (6)	C22—C23—N3—C24	177.7 (3)
C20—C21—C22—C23	0.0 (6)	C20—C19—N3—C23	0.8 (5)
C21—C22—C23—N3	0.0 (5)	C20—C19—N3—C24	-177.3 (3)
N3—C24—C25—C26	171.7 (3)	C25—C24—N3—C23	-112.1 (3)
C24—C25—C26—C27	-70.2 (4)	C25—C24—N3—C19	65.9 (4)
N4—C28—C29—C30	0.6 (5)	C31—C32—N4—C28	0.1 (5)
C28—C29—C30—C31	-0.7 (6)	C31—C32—N4—C33	179.7 (3)
C29—C30—C31—C32	0.5 (6)	C29—C28—N4—C32	-0.3 (5)
C30—C31—C32—N4	-0.2 (5)	C29—C28—N4—C33	-179.9 (3)
N4—C33—C34—C35	-68.6 (4)	C34—C33—N4—C32	106.5 (4)
C33—C34—C35—C36	-177.1 (3)	C34—C33—N4—C28	-73.9 (4)

Table S5. Geometrical data for the hydrogen bonds of **IL 13**.

C-H…Cl	d(H…Cl) [Å]	d(C…Cl) [Å]	∠(C-H…Cl) [°]	∠(H…Cl-M) [°]	Θ_t(C-H…Cl-M) [Å]
C2^{I}-H2^{I}…Cl1	2.8634(8)	3.576(4)	133.5(2)	123.97(3)	-2.1(3)
C32^{II}-H32A^{II}…Cl1	2.8124(9)	3.712(4)	161.1(2)	81.54(3)	173.3(6)
C19^{III}-H19^{III}…Cl3	2.8387(9)	3.628(4)	142.4(2)	81.89(3)	126.7(3)
C24^{III}-H24A^{III}…Cl3	2.8540(9)	3.627(4)	136.4(2)	133.4(4)	-55.9(3)
C15-H15B…Cl4	2.7151(9)	3.675(3)	166.2(2)	112.12(3)	-45.4(8)
C25-H25B…Cl4	2.8788(9)	3.756(4)	149.4(2)	81.40(3)	142.2(4)
C12^{IV}-H12^{IV}…Cl4	2.7526(7)	3.616(3)	152.9(2)	85.13(3)	-178.6(5)
C15-H15B…Cl5	2.8333(9)	3.631(4)	139.0(0)	131.78(4)	64.5(3)
C24-H24B…Cl6	2.7855(8)	3.729(3)	161.9(2)	119.93(4)	3.6(6)
C5^{V}-H5^{V} …Cl7	2.7978(9)	3.700(4)	161.2(2)	80.18(3)	-154.5(6)
C21^{IV}-H21^{IV}…Cl8	2.8326(8)	3.722(3)	158.2(2)	88.29(3)	177.3(6)

Symmetry operators: ^{I} 0.5+x. 1.5-y. -0.5+z ^{II} -0.5-x. -0.5+y. 0.5-z ^{III} 0.5-x. -0.5+y. 0.5-z
^{IV} –x. 2-y. 1-z ^{V} -0.5-x. 0.5+y. 0.5-z

$(C_4Py)_2[Cu_{0.41}Mn_{0.59}Cl_4]$ (IL 2)

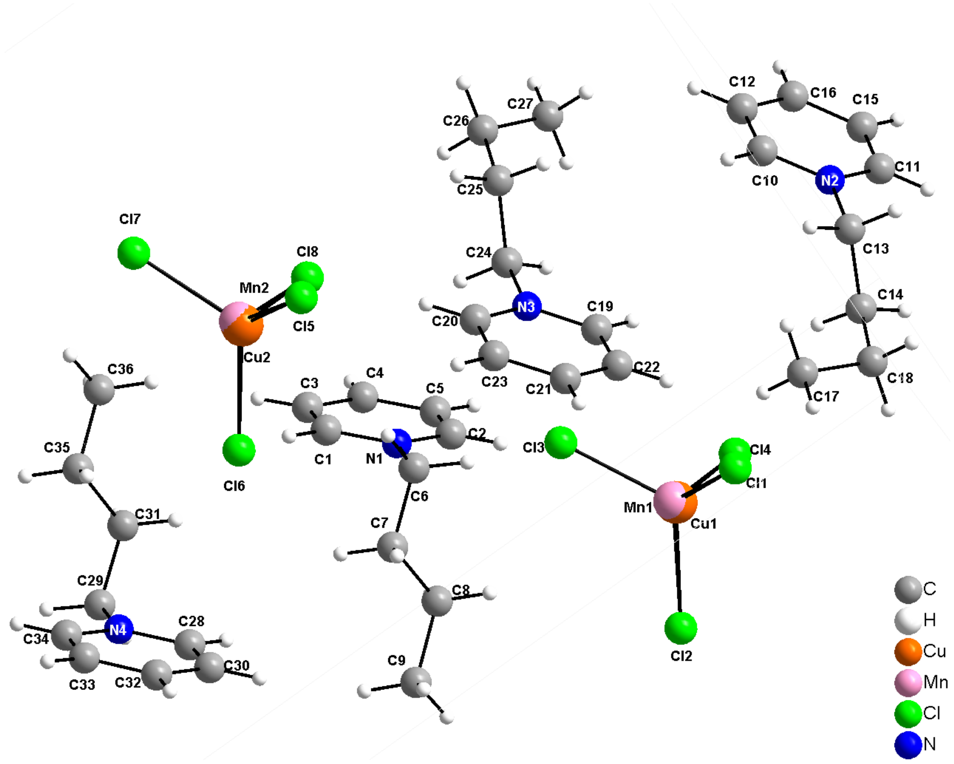

Figure S 3. Asymmetric unit of IL 2. For better overview the hydrogen labels are excluded.

Table S6. Bond lengths [Å] of **IL 2**.

Bond	d [Å]	Bond	d [Å]	Bond	d [Å]
C1—N1	1.336 (5)	C14—H14B	0.9800	C29—C31	1.512 (6)
C1—C3	1.372 (7)	C15—C16	1.372 (7)	C29—H29A	0.9800
C1—H1	0.9400	C15—H15	0.9400	C29—H29B	0.9800
C2—N1	1.347 (6)	C16—H16	0.9400	C30—C32	1.378 (7)
C2—C5	1.361 (8)	C17—C18	1.521 (7)	C30—H30	0.9400
C2—H2	0.9400	C17—H17A	0.9700	C31—C35	1.528 (6)
C3—C4	1.386 (8)	C17—H17B	0.9700	C31—H31A	0.9800
C3—H3	0.9400	C17—H17C	0.9700	C31—H31B	0.9800
C4—C5	1.384 (7)	C18—H18A	0.9800	C32—C33	1.371 (7)
C4—H4	0.9400	C18—H18B	0.9800	C32—H32	0.9400
C5—H5	0.9400	C19—N3	1.342 (5)	C33—C34	1.373 (8)
C6—N1	1.493 (6)	C19—C22	1.360 (6)	C33—H33	0.9400
C6—C7	1.505 (7)	C19—H19	0.9400	C34—N4	1.342 (6)
C6—H6A	0.9800	C20—N3	1.345 (6)	C34—H34	0.9400
C6—H6B	0.9800	C20—C23	1.367 (6)	C35—C36	1.522 (7)
C7—C8	1.452 (8)	C20—H20	0.9400	C35—H35A	0.9800
C7—H7A	0.9800	C21—C23	1.371 (6)	C35—H35B	0.9800
C7—H7B	0.9800	C21—C22	1.376 (7)	C36—H36A	0.9700
C8—C9	1.563 (8)	C21—H21	0.9400	C36—H36B	0.9700
C8—H8A	0.9800	C22—H22	0.9400	C36—H36C	0.9700
C8—H8B	0.9800	C23—H23	0.9400	Cu1—Cl2	2.281 (5)
C9—H9A	0.9700	C24—N3	1.486 (6)	Cu1—Cl4	2.290 (6)
C9—H9B	0.9700	C24—C25	1.532 (7)	Cu1—Cl3	2.352 (6)
C9—H9C	0.9700	C24—H24A	0.9800	Cu1—Cl1	2.365 (6)
C10—N2	1.338 (5)	C24—H24B	0.9800	Mn1—Cl3	2.287 (4)
C10—C12	1.369 (7)	C25—C26	1.519 (7)	Mn1—Cl2	2.313 (3)
C10—H10	0.9400	C25—H25A	0.9800	Mn1—Cl1	2.324 (4)
C11—N2	1.344 (6)	C25—H25B	0.9800	Mn1—Cl4	2.368 (4)
C11—C15	1.359 (6)	C26—C27	1.463 (8)	Cu2—Cl6	2.272 (7)
C11—H11	0.9400	C26—H26A	0.9800	Cu2—Cl5	2.327 (7)
C12—C16	1.368 (7)	C26—H26B	0.9800	Cu2—Cl8	2.335 (6)
C12—H12	0.9400	C27—H27A	0.9700	Cu2—Cl7	2.351 (8)
C13—N2	1.500 (5)	C27—H27B	0.9700	Mn2—Cl7	2.265 (6)
C13—C14	1.523 (6)	C27—H27C	0.9700	Mn2—Cl6	2.334 (5)
C13—H13A	0.9800	C28—N4	1.340 (5)	Mn2—Cl5	2.340 (5)
C13—H13B	0.9800	C28—C30	1.369 (7)	Mn2—Cl8	2.347 (4)
C14—C18	1.508 (7)	C28—H28	0.9400		
C14—H14A	0.9800	C29—N4	1.492 (6)		

Table S7. Bond angles [°] of **IL 2**.

Bond angle	∠ [°]	Bond angle	∠ [°]	Bond angle	∠ [°]
N1—C1—C3	120.8 (5)	C12—C16—H16	120.2	C32—C30—H30	120.0
N1—C1—H1	119.6	C15—C16—H16	120.2	C29—C31—C35	113.7 (4)
C3—C1—H1	119.6	C18—C17—H17A	109.5	C29—C31—H31A	108.8
N1—C2—C5	121.0 (5)	C18—C17—H17B	109.5	C35—C31—H31A	108.8
N1—C2—H2	119.5	H17A—C17—H17B	109.5	C29—C31—H31B	108.8
C5—C2—H2	119.5	C18—C17—H17C	109.5	C35—C31—H31B	108.8
C1—C3—C4	119.5 (5)	H17A—C17—H17C	109.5	H31A—C31—H31B	107.7
C1—C3—H3	120.2	H17B—C17—H17C	109.5	C33—C32—C30	119.1 (5)
C4—C3—H3	120.2	C14—C18—C17	113.0 (4)	C33—C32—H32	120.5
C5—C4—C3	118.6 (5)	C14—C18—H18A	109.0	C30—C32—H32	120.5
C5—C4—H4	120.7	C17—C18—H18A	109.0	C32—C33—C34	119.3 (5)
C3—C4—H4	120.7	C14—C18—H18B	109.0	C32—C33—H33	120.3
C2—C5—C4	119.6 (5)	C17—C18—H18B	109.0	C34—C33—H33	120.3
C2—C5—H5	120.2	H18A—C18—H18B	107.8	N4—C34—C33	120.8 (4)
C4—C5—H5	120.2	N3—C19—C22	120.7 (4)	N4—C34—H34	119.6
N1—C6—C7	113.7 (4)	N3—C19—H19	119.7	C33—C34—H34	119.6
N1—C6—H6A	108.8	C22—C19—H19	119.7	C36—C35—C31	111.6 (4)
C7—C6—H6A	108.8	N3—C20—C23	120.3 (4)	C36—C35—H35A	109.3
N1—C6—H6B	108.8	N3—C20—H20	119.8	C31—C35—H35A	109.3
C7—C6—H6B	108.8	C23—C20—H20	119.8	C36—C35—H35B	109.3
H6A—C6—H6B	107.7	C23—C21—C22	119.5 (5)	C31—C35—H35B	109.3
C8—C7—C6	113.9 (5)	C23—C21—H21	120.2	H35A—C35—H35B	108.0
C8—C7—H7A	108.8	C22—C21—H21	120.2	C35—C36—H36A	109.5
C6—C7—H7A	108.8	C19—C22—C21	119.4 (4)	C35—C36—H36B	109.5
C8—C7—H7B	108.8	C19—C22—H22	120.3	H36A—C36—H36B	109.5
C6—C7—H7B	108.8	C21—C22—H22	120.3	C35—C36—H36C	109.5
H7A—C7—H7B	107.7	C20—C23—C21	119.5 (5)	H36A—C36—H36C	109.5
C7—C8—C9	111.9 (5)	C20—C23—H23	120.3	H36B—C36—H36C	109.5
C7—C8—H8A	109.2	C21—C23—H23	120.3	Cl2—Cu1—Cl4	106.8 (2)
C9—C8—H8A	109.2	N3—C24—C25	109.6 (4)	Cl2—Cu1—Cl3	119.8 (2)
C7—C8—H8B	109.2	N3—C24—H24A	109.8	Cl4—Cu1—Cl3	107.5 (2)
C9—C8—H8B	109.2	C25—C24—H24A	109.8	Cl2—Cu1—Cl1	108.8 (2)
H8A—C8—H8B	107.9	N3—C24—H24B	109.8	Cl4—Cu1—Cl1	111.8 (2)
C8—C9—H9A	109.5	C25—C24—H24B	109.8	Cl3—Cu1—Cl1	102.1 (2)
C8—C9—H9B	109.5	H24A—C24—H24B	108.2	Cl3—Mn1—Cl2	121.27 (19)
H9A—C9—H9B	109.5	C26—C25—C24	113.6 (5)	Cl3—Mn1—Cl1	105.44 (13)
C8—C9—H9C	109.5	C26—C25—H25A	108.8	Cl2—Mn1—Cl1	109.13 (16)
H9A—C9—H9C	109.5	C24—C25—H25A	108.8	Cl3—Mn1—Cl4	107.09 (15)
H9B—C9—H9C	109.5	C26—C25—H25B	108.8	Cl2—Mn1—Cl4	103.22 (13)
N2—C10—C12	120.5 (4)	C24—C25—H25B	108.8	Cl1—Mn1—Cl4	110.54 (17)
N2—C10—H10	119.8	H25A—C25—H25B	107.7	Cl6—Cu2—Cl5	108.5 (3)
C12—C10—H10	119.8	C27—C26—C25	113.8 (5)	Cl6—Cu2—Cl8	106.9 (3)
N2—C11—C15	120.7 (4)	C27—C26—H26A	108.8	Cl5—Cu2—Cl8	111.5 (2)
N2—C11—H11	119.7	C25—C26—H26A	108.8	Cl6—Cu2—Cl7	122.1 (3)
C15—C11—H11	119.7	C27—C26—H26B	108.8	Cl5—Cu2—Cl7	102.9 (3)
C16—C12—C10	119.3 (4)	C25—C26—H26B	108.8	Cl8—Cu2—Cl7	104.9 (3)
C16—C12—H12	120.4	H26A—C26—H26B	107.7	Cl7—Mn2—Cl6	123.2 (2)
C10—C12—H12	120.4	C26—C27—H27A	109.5	Cl7—Mn2—Cl5	105.18 (17)
N2—C13—C14	110.8 (4)	C26—C27—H27B	109.5	Cl6—Mn2—Cl5	105.9 (2)
N2—C13—H13A	109.5	H27A—C27—H27B	109.5	Cl7—Mn2—Cl8	107.3 (2)
C14—C13—H13A	109.5	C26—C27—H27C	109.5	Cl6—Mn2—Cl8	104.42 (17)
N2—C13—H13B	109.5	H27A—C27—H27C	109.5	Cl5—Mn2—Cl8	110.6 (2)
C14—C13—H13B	109.5	H27B—C27—H27C	109.5	C1—N1—C2	120.4 (4)
H13A—C13—H13B	108.1	N4—C28—C30	120.3 (4)	C1—N1—C6	118.6 (4)
C18—C14—C13	112.8 (4)	N4—C28—H28	119.9	C2—N1—C6	121.0 (4)
C18—C14—H14A	109.0	C30—C28—H28	119.9	C10—N2—C11	120.5 (4)
C13—C14—H14A	109.0	N4—C29—C31	111.9 (4)	C10—N2—C13	119.7 (4)
C18—C14—H14B	109.0	N4—C29—H29A	109.2	C11—N2—C13	119.7 (3)
C13—C14—H14B	109.0	C31—C29—H29A	109.2	C19—N3—C20	120.6 (4)
H14A—C14—H14B	107.8	N4—C29—H29B	109.2	C19—N3—C24	119.8 (4)
C11—C15—C16	119.3 (5)	C31—C29—H29B	109.2	C20—N3—C24	119.6 (4)
C11—C15—H15	120.3	H29A—C29—H29B	107.9	C28—N4—C34	120.6 (4)
C16—C15—H15	120.3	C28—C30—C32	119.9 (4)	C28—N4—C29	118.2 (4)
C12—C16—C15	119.7 (5)	C28—C30—H30	120.0	C34—N4—C29	121.2 (4)

Table S8. Torsion angles [°] of **IL 2**.

Torsion angle	∠ [°]	Torsion angle	∠ [°]
N1—C1—C3—C4	-0.7 (7)	C3—C1—N1—C2	0.7 (6)
C1—C3—C4—C5	0.5 (8)	C3—C1—N1—C6	-179.9 (4)
N1—C2—C5—C4	0.2 (8)	C5—C2—N1—C1	-0.4 (7)
C3—C4—C5—C2	-0.3 (8)	C5—C2—N1—C6	-179.8 (4)
N1—C6—C7—C8	68.8 (6)	C7—C6—N1—C1	74.1 (5)
C6—C7—C8—C9	176.6 (5)	C7—C6—N1—C2	-106.5 (5)
N2—C10—C12—C16	-0.1 (7)	C12—C10—N2—C11	-0.3 (7)
N2—C13—C14—C18	-173.3 (4)	C12—C10—N2—C13	-177.2 (4)
N2—C11—C15—C16	0.4 (7)	C15—C11—N2—C10	0.1 (7)
C10—C12—C16—C15	0.6 (8)	C15—C11—N2—C13	177.1 (4)
C11—C15—C16—C12	-0.8 (8)	C14—C13—N2—C10	111.8 (4)
C13—C14—C18—C17	69.1 (6)	C14—C13—N2—C11	-65.2 (5)
N3—C19—C22—C21	0.8 (7)	C22—C19—N3—C20	-1.8 (6)
C23—C21—C22—C19	0.2 (7)	C22—C19—N3—C24	175.6 (4)
N3—C20—C23—C21	-0.5 (7)	C23—C20—N3—C19	1.6 (6)
C22—C21—C23—C20	-0.4 (7)	C23—C20—N3—C24	-175.7 (4)
N3—C24—C25—C26	176.1 (4)	C25—C24—N3—C19	-104.4 (5)
C24—C25—C26—C27	-74.9 (7)	C25—C24—N3—C20	73.0 (5)
N4—C28—C30—C32	0.3 (7)	C30—C28—N4—C34	0.3 (6)
N4—C29—C31—C35	-68.5 (5)	C30—C28—N4—C29	-179.8 (4)
C28—C30—C32—C33	-1.0 (7)	C33—C34—N4—C28	-0.2 (7)
C30—C32—C33—C34	1.2 (8)	C33—C34—N4—C29	179.9 (4)
C32—C33—C34—N4	-0.6 (8)	C31—C29—N4—C28	-75.4 (5)
C29—C31—C35—C36	-175.5 (4)	C31—C29—N4—C34	104.5 (5)

Table S9. Geometrical data for the hydrogen bonds of **IL 2**.

C-H…Cl	d(H…Cl) [Å]	d(C…Cl) [Å]	∠(C-H…Cl) [°]	∠(H…Cl-M) [°]	Θ_t(C-H…Cl-M) [Å]
$C2^{I}$-$H2^{I}$…Cl1	2.8741(12)	3.573(5)	132.1(3)	126.98(16)	-7.6(5)
$C30^{II}$-$H30^{II}$…Cl1	2.8209(12)	3.718(7)	159.8(4)	78.25(15)	176.6(11)
$C32^{II}$-$H32^{II}$…Cl2	2.8549(12)	3.738(5)	157.0(3)	91.07(16)	136.9(9)
C21-H21…Cl3	2.7542(11)	3.624(4)	154.1(3)	86.38(16)	-174.8(7)
$C24^{I}$-$H24B^{I}$…Cl3	2.7206(15)	3.681(5)	166.2(3)	117.88(17)	-34.0(13)
$C11^{III}$-$H11^{III}$…Cl4	2.80743(11)	3.652(5)	140.8(3)	78.71(16)	126.1(5)
$C13^{III}$-$H13B^{III}$…Cl4	2.8377(12)	3.619(4)	137.3(2)	130.53(16)	-53.8(4)
$C12^{I}$-$H12^{I}$…Cl5	2.8689(11)	3.564(5)	131.6(3)	127.73(20)	15.2(5)
$C34^{IV}$-$H34^{IV}$…Cl5	2.8160(12)	3.716(6)	160.6(3)	78.19(18)	160.8(11)
$C16^{I}$-$H16^{I}$…Cl6	2.8047(12)	3.701(5)	159.7(3)	90.39(19)	-172.3(10)
$C13^{IV}$-$H13A^{IV}$…Cl7	2.7910(15)	3.729(5)	160.2(3)	125.31(18)	6.0(8)
C24-H24…Cl8	2.8049(12)	3.614(6)	140.3(3)	130.11(14)	-61.7(5)

Symmetry operators: ^{I} -0.5+x. 0.5-y. -0.5+z ^{II} 0.5-x. -0.5+y. 0.5-z ^{III} 1-x. -y. 1-z
^{IV} 0.5-x. -0.5+y. 1.5-z

3. TG and DSC measurements

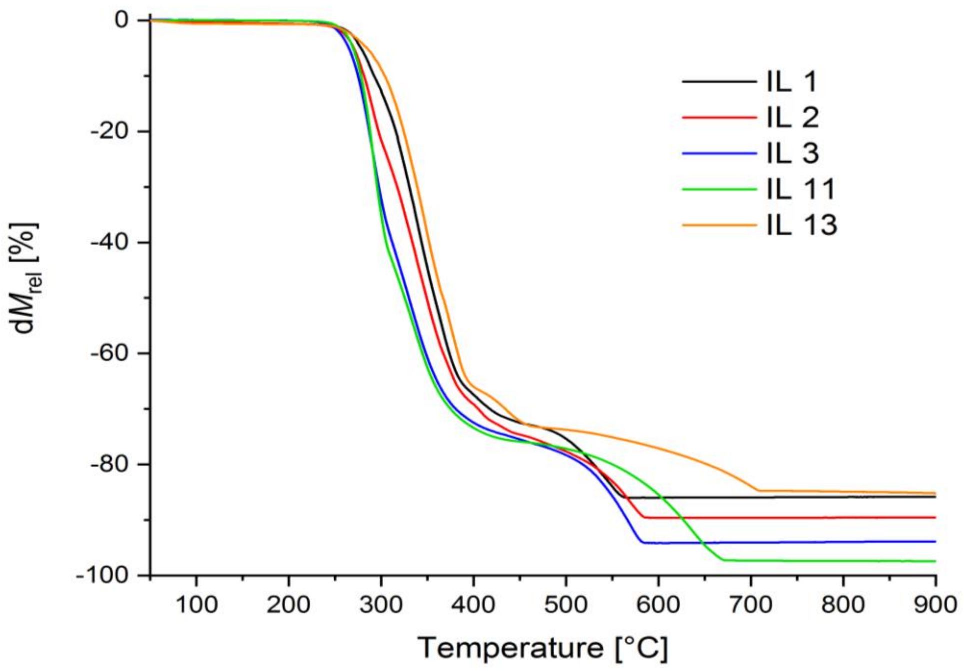

Figure S04. TGA data of the copper-IL (IL 11), the manganese-IL (IL 13), and the copper-manganese-ILs (IL 1-3).

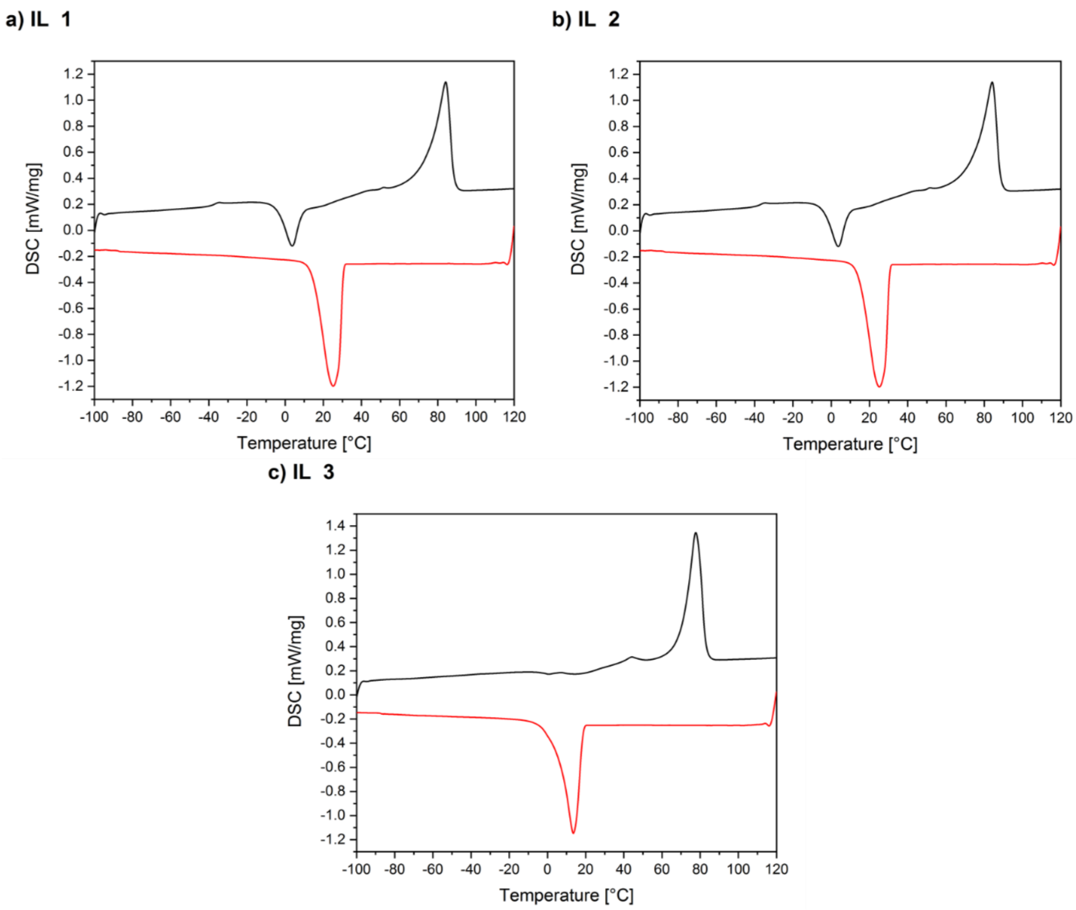

Figure S 5. DSC data of ILs 1-3. Second cooling and heating cycles are shown.

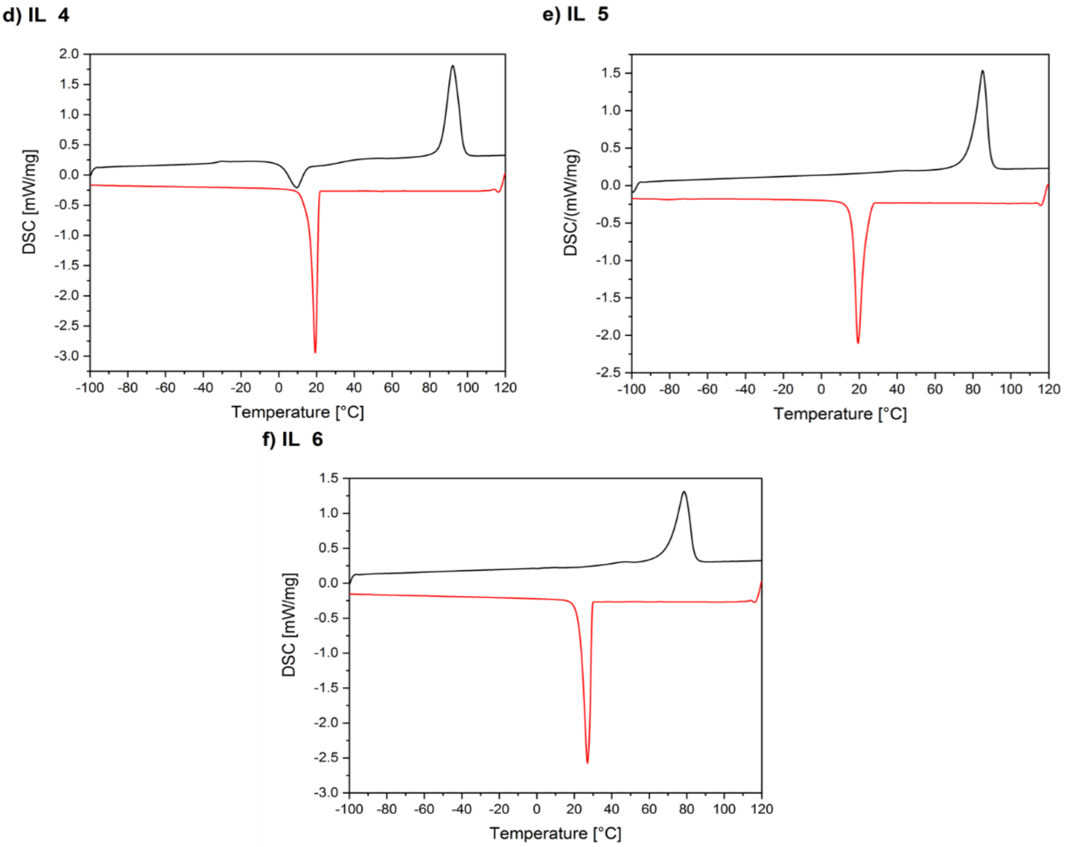

Figure S 6. DSC data of ILs 4-6. Second cooling and heating cycles are shown.

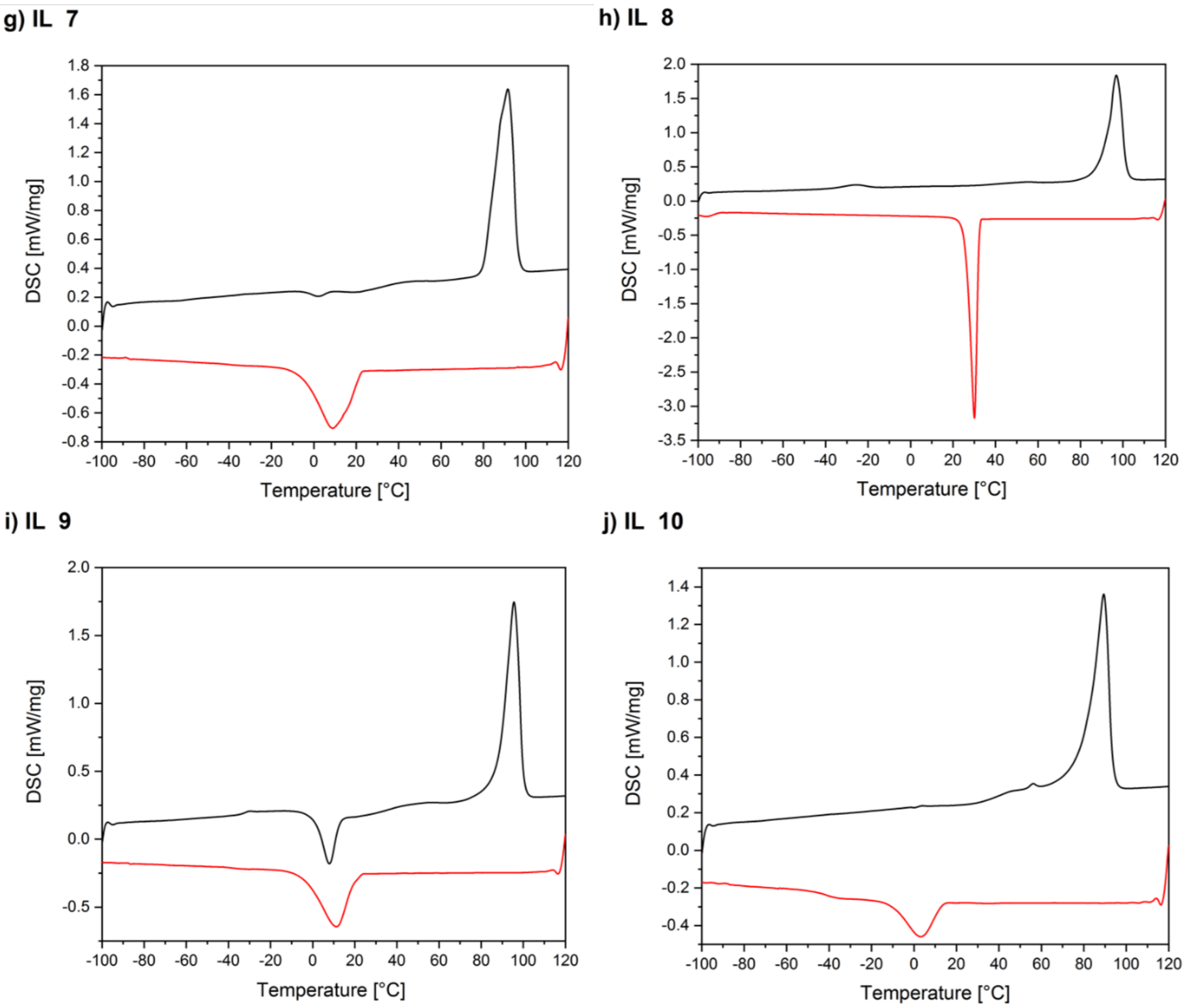

Figure S 7. DSC data of ILs 7-10. Second cooling and heating cycles are shown.

Table S10. Melting transitions extracted from DSC heating curves (IL1-10).[a,b]

Compound	Run	T_m [°C]	ΔH [kJ/mol]
**IL1**	2nd	79.6	28.70
	3rd	80.7	27.88
**IL2**	2nd	73.2	25.42
	3rd	73.5	25.44
**IL3**	2nd	69.8	26.54
	3rd	70.0	26.56
**IL4**	2nd	85.8	33.02
	3rd	86.6	33.02
**IL5**	2nd	77.7	29.89
	3rd	76.7	30.45
**IL6**	2nd	69.5	28.25
	3rd	69.6	28.33
**IL7**	2nd	80.6	33.35
	3rd	79.4	33.06
**IL8**	2nd	92.6	35.19
	3rd	92.8	35.14
**IL9**	2nd	87.5	33.50
	3rd	88.0	33.48
**IL10**	2nd	79.9	28.03
	3rd	80.4	27.80
**IL11**	2nd	-*	-*
	3rd	67.0*	4.28*
**IL12**	2nd	63.8	28.90
	3rd	69.3	30.52
**IL13**	2nd	76.8	28.51
	3rd	85.3	25.78

[a] DSC data from **IL11-13** are consistent with published data.[10] [b] The first heating-cooling-cycle is not shown. ***IL11** does not show a clean melting point in the second and third heating cycle.

4. CV- and Impedance measurements

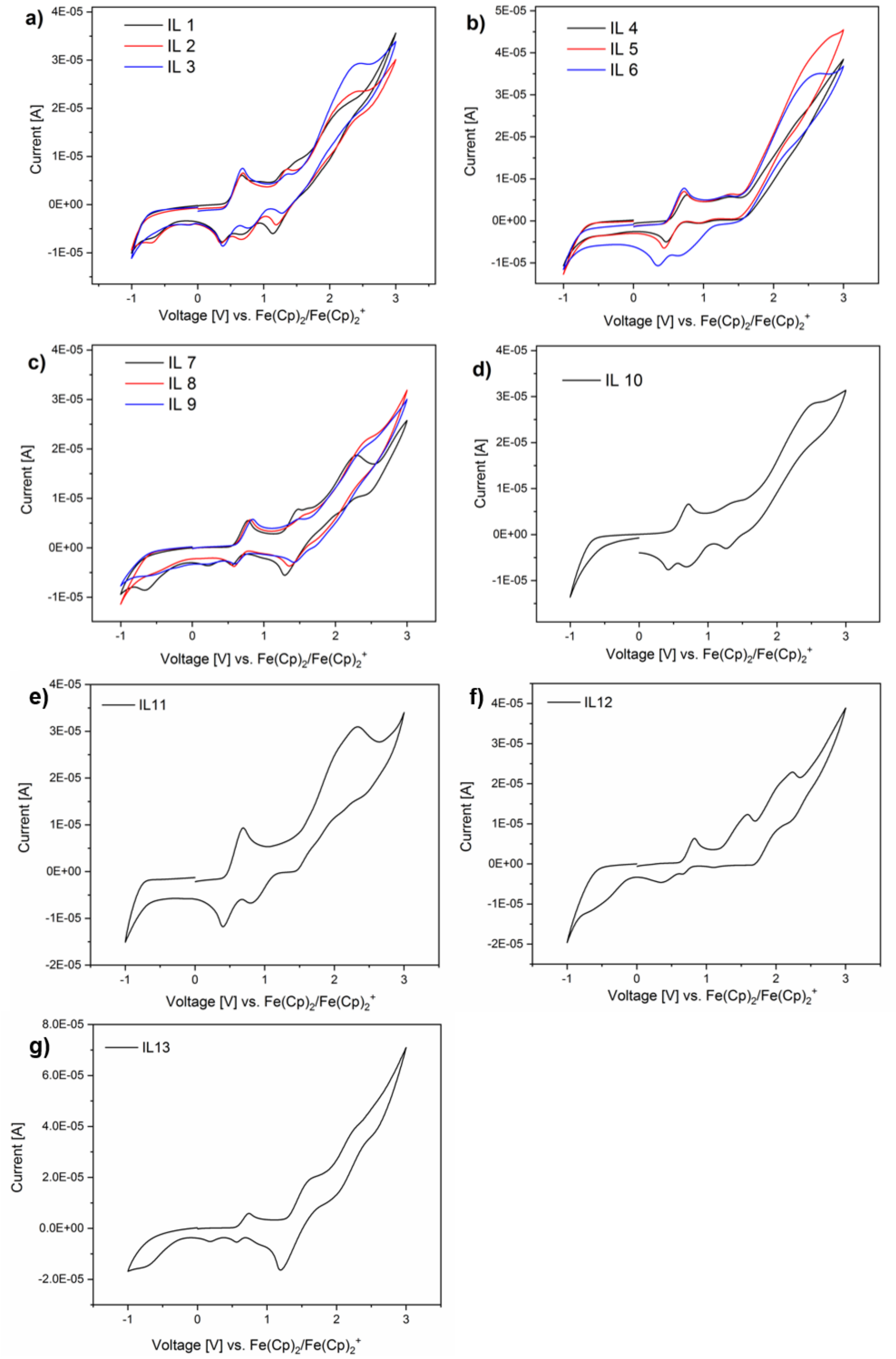

Figure S 8. Cyclovoltammogram of a) the Copper-Manganese-ILs, b) Copper-Cobalt-ILs, c) Cobalt-Manganese-ILs, d) the trimetallic ILs, e) the Copper-IL, f) the Cobalt-IL and g) the Manganese-IL.

Table S11. Impedance-data with the equivalent circuit diagrams. Data was evaluated using rhd software RelaxIS®.

Compound	Temperature [°C]	Resistance [Ω]	Conductivity [S cm^{-1}]	Equivalent Circuits
**IL 1**	30	4.57E+04 ± 34	2.48E-04	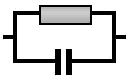
	40	1.18E+04 ± 13	9.55E-04	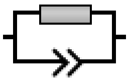
	50	3230.2 ± 15.7	3.50E-03	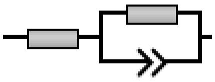
	60	772.63 ± 2.24	1.46E-02	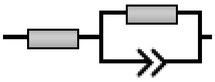
	70	328.88 ± 4.13	3.44E-02	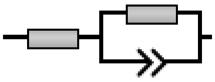
Compound	Temperature [°C]	Resistance [Ω]	Conductivity [S cm^{-1}]	Equivalent Circuits
**IL 2**	30	4.51E+05 ± 686	2.09E-05	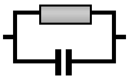
	40	8.55E+04 ± 308	1.10E-04	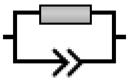
	50	10914 ± 24.1	8.64E-04	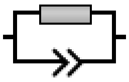
	60	2.30E+02 ± 13.3	4.10E-02	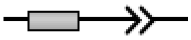
	70	64.081 ± 57.2	1.47E-01	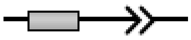
Compound	Temperature [°C]	Resistance [Ω]	Conductivity [S cm^{-1}]	Equivalent Circuits
**IL 3**	30	82001 ± 424	1.53E-04	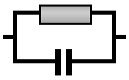
	40	42190 ± 130	2.98E-04	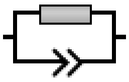
	50	43849 ± 478	2.87E-04	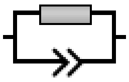
	60	95799 ± 2145	1.31E-04	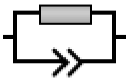
	70	1.75E+05 ± 3719	7.19E-05	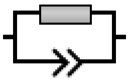

Compound	Temperature [°C]	Resistance [Ω]	Conductivity [S cm^{-1}]	Equivalent Circuits
**IL 4**	30	351.71 ± 0.575	3.06E-02	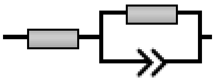
	40	242.47 ± 0.146	4.44E-02	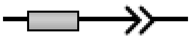
	50	209.82 ± 0.267	5.13E-02	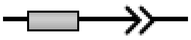
	60	237.61 ± 0.127	4.53E-02	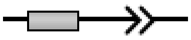
	70	433.16 ± 2.17	2.49E-02	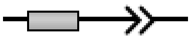
Compound	Temperature [°C]	Resistance [Ω]	Conductivity [S cm^{-1}]	Equivalent Circuits
**IL 5**	30	7.35E+05 ± 2030	1.03E-05	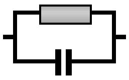
	40	1.64E+05 ± 904	4.60E-05	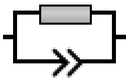
	50	37314 ± 74.4	2.02E-04	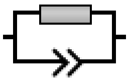
	60	8957.1 ± 19.7	8.42E-04	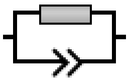
	70	2501.4 ± 5.66	3.01E-03	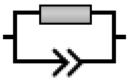
Compound	Temperature [°C]	Resistance [Ω]	Conductivity [S cm^{-1}]	Equivalent Circuits
**IL 6**	30	6.96E+05 ± 3989	1.41E-05	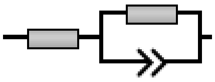
	40	1.84E+05 ± 444	5.35E-05	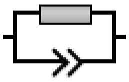
	50	35706 ± 28	2.75E-04	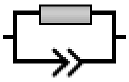
	60	3764.4 ± 6.19	2.61E-03	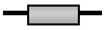
	70	237.13 ± 1.94	4.15E-02	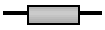
Compound	Temperature [°C]	Resistance [Ω]	Conductivity [S cm^{-1}]	Equivalent Circuits
**IL 7**	30	50004 ± 93.1	1.26E-04	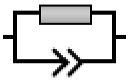
	40	22744 ± 67.5	2.76E-04	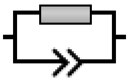
	50	10790 ± 21.9	5.82E-04	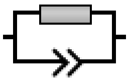
	60	5257.8 ± 9.2	1.20E-03	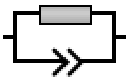
	70	850.36 ± 19	7.39E-03	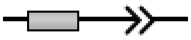

Compound	Temperature [°C]	Resistance [Ω]	Conductivity [S cm^{-1}]	Equivalent Circuits
**IL 8**	30	31447 ± 64.4	2.66E-04	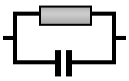
	40	20810 ± 60.6	4.03E-04	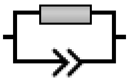
	50	12265 ± 23.2	6.83E-04	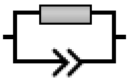
	60	5582.5 ± 8.84	1.50E-03	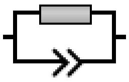
	70	2244 ± 4.16	3.73E-03	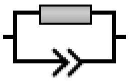
Compound	Temperature [°C]	Resistance [Ω]	Conductivity [S cm^{-1}]	Equivalent Circuits
**IL 9**	30	2.06E+08 ± 1.55E+07	5.23E-08	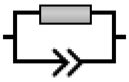
	40	8.39E+07 ± 7.27E+06	1.28E-07	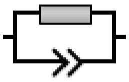
	50	4.13E+07 ± 1.84E+06	2.61E-07	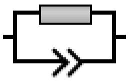
	60	1.30E+07 ± 2.09E+05	8.29E-07	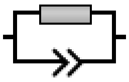
	70	2.28E+06 ± 7392	4.72E-06	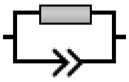
Compound	Temperature [°C]	Resistance [Ω]	Conductivity [S cm^{-1}]	Equivalent Circuits
**IL 10**	30	21236 ± 56.3	4.63E-04	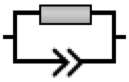
	40	7242 ± 22.4	1.36E-03	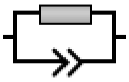
	50	2118.3 ± 2.22	4.64E-03	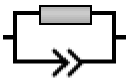
	60	540.58 ± 1.07	1.82E-02	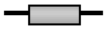
	70	215.17 ± 4.45	4.57E-02	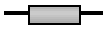

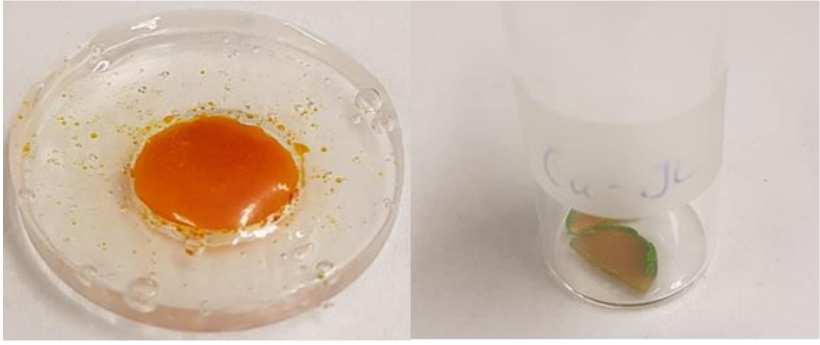

Figure S 9. Photographs of IL11. Left: Before conductivity measurement. Right: After conductivity measurement.

5. SEM and EDX measurements

Spectrum processing :
Peak possibly omitted : 8.710 keV

Processing option : All elements analyzed (Normalised)
Number of iterations = 3

Standard :
O SiO2 1-Jun-1999 12:00 AM
Na Albite 1-Jun-1999 12:00 AM
Al Al2O3 1-Jun-1999 12:00 AM
S FeS2 1-Jun-1999 12:00 AM
Cl KCl 1-Jun-1999 12:00 AM
Ca Wollastonite 1-Jun-1999 12:00 AM
Mn Mn 1-Jun-1999 12:00 AM
Ni Ni 1-Jun-1999 12:00 AM

Element	Weight%	Atomic%
O K	15.98	31.93
Na K	1.07	1.49
Al K	31.32	37.11
S K	0.70	0.70
Cl K	2.39	2.16
Ca K	0.58	0.46
Mn K	0.95	0.55
Ni K	47.02	25.60
Totals	100.00	

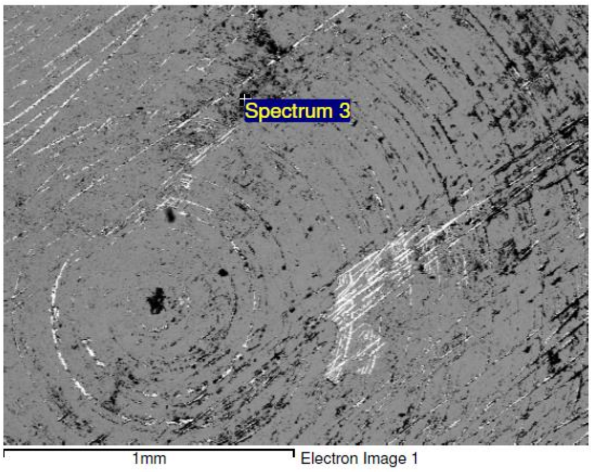

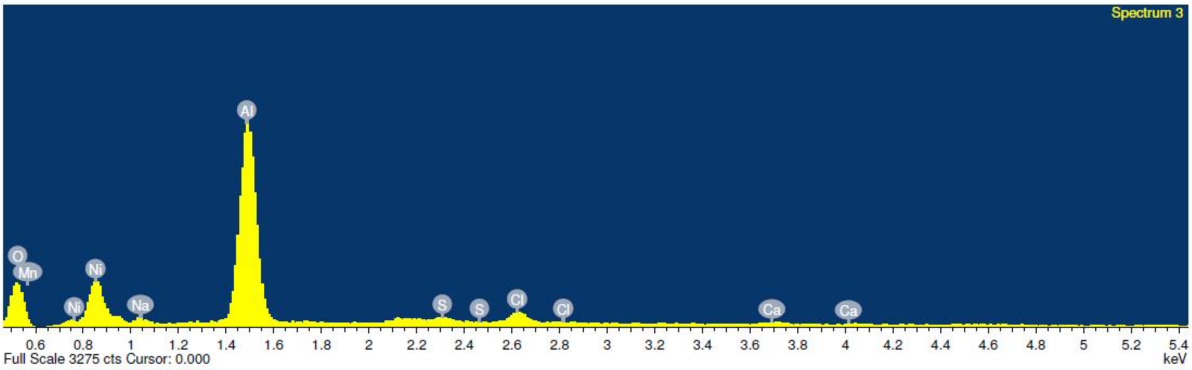

Figure S 10. EDX data of a gold electrode after the measurement of IL13.

6. UV/Vis measurements

Table S12. Direct optical band gaps of **IL 1-9** obtained from the Tauc plot analysis.

Compound	Direct optical band gap [eV]	Compound	Direct optical band gap [eV]
**IL1**	4.39 ± 0.02	**IL6**	4.32 ± 0.02
**IL2**	4.38 ± 0.02	**IL7**	4.49 ± 0.02
**IL3**	4.38 ± 0.02	**IL8**	4.40 ± 0.02
**IL4**	4.40 ± 0.02	**IL9**	4.39 ± 0.02
**IL5**	4.34 ± 0.02		

7. General Synthesis

Table S13. Mass Calculation and Yields of the synthesized ILs.

IL	m(BuPyCl) [mg]	m($CuCl_2$) [mg]	m($CoCl_2$) [mg]	m($MnCl_2$) [mg]	m(IL) [mg]	Yield [%]
**1**	205	26	-	75	269	96
**2**	204	54	-	53	262	93
**3**	199	77	-	24	250	91
**4**	201	25	105	-	258	93
**5**	200	55	70	-	231	83
**6**	200	75	34	-	270	97
**7**	205	-	34	76	261	93
**8**	204	-	69	49	271	97
**9**	200	-	105	25	262	95
**10**	200	33	47	33	270	98
**11**	208	101	-	-	261	92
**12**	200	-	141	-	271	97
**13**	203	-	-	980	248	78
